# ECCENTRIC: a fast and unrestrained approach for high-resolution in vivo metabolic imaging at ultra-high field MR

**Published:** 2023-05-23

**Authors:** Antoine Klauser, Bernhard Strasser, Wolfgang Bogner, Lukas Hingerl, Sebastien Courvoisier, Claudiu Schirda, Mehran Baboli, Jorg Dietrich, Isabel Arrillaga-Romany, Julie Miller, Erik Uhlmann, Daniel P. Cahill, Tracy T. Batchelor, Francois Lazeyras, Ovidiu C. Andronesi

**Affiliations:** a Athinoula A. Martinos Center for Biomedical Imaging, Department of Radiology, Massachusetts General Hospital, Harvard Medical School, Boston; b Advanced Clinical Imaging Technology, Siemens Healthcare AG, Lausanne, Switzerland; c High-Field MR Center, Department of Biomedical Imaging and Image-guided Therapy, Medical University of Vienna, Vienna, Austria; d Department of Radiology, University of Pittsburgh School of Medicine, Pittsburgh, Pennsylvania, USA; e Department of Neurology, Massachusetts General Hospital, Harvard Medical School, Boston; f Department of Neurology, Beth Israel Deaconess Medical Center, Harvard Medical School, Boston; g Department of Neurosurgery, Massachusetts General Hospital, Harvard Medical School, Boston; h Department of Neurology, Brigham and Women, Harvard Medical School, Boston; i Department of Radiology and Medical Informatics, University of Geneva, Switzerland; j CIBM Center for Biomedical Imaging, Switzerland

## Abstract

A novel method for fast and high-resolution metabolic imaging, called ECcentric Circle ENcoding TRajectorIes for Compressed sensing (ECCENTRIC), has been developed and implemented on 7 Tesla human MRI. ECCENTRIC is a non-Cartesian spatial-spectral encoding method optimized for random undersampling of magnetic resonance spectroscopic imaging (MRSI) at ultra-high field. The approach provides flexible and random (k,t) sampling without temporal interleaving to improve spatial response function and spectral quality. ECCENTRIC needs low gradient amplitudes and slew-rates that reduces electrical, mechanical and thermal stress of the scanner hardware, and is robust to timing imperfection and eddy-current delays. Combined with a model-based low-rank reconstruction, this approach enables simultaneous imaging of up to 14 metabolites over the whole-brain at 2–3mm isotropic resolution in 4–10 minutes with high signal-to-noise ratio. In 20 healthy volunteers and 20 glioma patients ECCENTRIC demonstrated unprecedented mapping of fine structural details of metabolism in healthy brains and an extended metabolic fingerprinting of glioma tumors.

## I. INTRODUCTION

Magnetic resonance spectroscopic imaging (MRSI) is a molecular MR imaging modality that can be used to investigate *in vivo* metabolism in humans non-invasively with non-ionizing radiation. In particular, ^1^H-MRSI can simultaneously image up to 20 metabolites in the brain and assess their concentration [1] and the dynamic change of their concentrations under functional tasks [2, 3]. MRSI is capable of measuring intrinsic metabolism without the need of contrast agents, and can probe metabolic enzymatic rates that are not accessible by radio-tracer imaging techniques such as PET and SPECT [4]. Numerous studies have demonstrated the considerable potential of MRSI for clinical applications, but the performance of current MRSI protocols limits their use in routine clinical investigations [5], which is severely lacking behind other MR modalities.

MRSI at ultra-high field (UHF ≥ 7T) using very short echo time (≈ 1ms) free induction decay (FID) excitation is a method that is gaining large popularity due to its high SNR for metabolite imaging [6–8]. Nevertheless, a major drawback of MRSI is low resolution and long acquisition times of the 4D (k,t) spatial-temporal space, resulting in a critical need for imaging acceleration strategies [9]. This is particularly relevant for high-resolution whole-brain MRSI where traditional phase-encoding acquisition schemes would require several hours. Acceleration of UHF MRSI has been shown by parallel imaging such as SENSE, GRAPPA and CAIPIRINHA with uniform undersampling [10–12], or by Compressed Sensing (CS) with random undersampling [13], but these techniques generally don’t allow acceleration factors (AF) above 6–10 for MRSI. Additionally, spatial-spectral encoding (SSE) techniques enable further acceleration for UHF MRSI. By combining spatial-spectral encoding with undersampling higher accelerations of UHF MRSI may be achieved [14–16].

So far, SSE has been demonstrated at ultra-high fields using either Cartesian (EPSI) [17–19] and non-Cartesian (spirals, rosettes, concentric circles) [14, 20–22] k-space trajectories. Despite their potential benefits, these methods have an important limitation that requires the use of temporal interleaves to achieve broad spectral bandwidth and high spatial resolution. As the field strength increases, this becomes even more challenging. Temporal interleaving prolongs the acquisition time and creates spectral side-bands from spurious signals that may overlap with metabolite peaks, ultimately degrading the spectral quality [9].

In the current work, we sought to significantly improve metabolic imaging by addressing limitations of the current methods in order to maximize the acceleration, spectral quality and flexibility for high-resolution 4D (k,t) sampling at ultra-high field. Our first guiding principle was to obtain advanced metabolic imaging that is feasible and robust for clinical use. In addition, pushing the spatial-temporal limits of metabolic imaging for state-of-the-art research protocols formed our second line of thought in the overall concept.

**FIG. 1:**
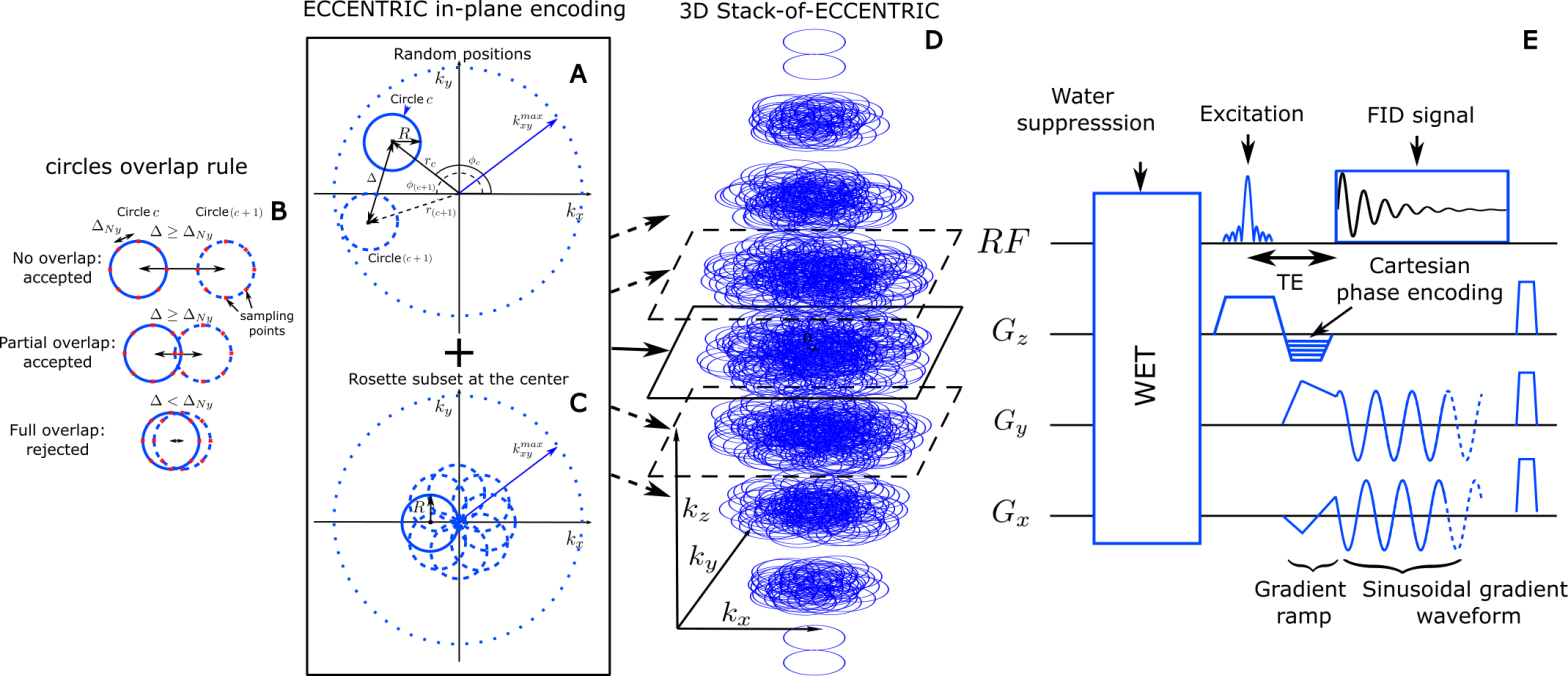
(A) circle center positions are parameterized in polar coordinates $\left(r_{c},\phi_{c}\right)$ that are chosen randomly in the ranges $r_{c}\in \left[0,max\left(k_{xy}^{max}-r,r\right)\right]$ and $\phi_{c}\in [0,2\pi ]$. Two consecutive circles (c and c+1) are shown and must respect the overlap rule described in (B): the distance between their respective centers, Δ, must be greater or equal to the Nyquist distance, $\Delta_{Ny}$. (C), to satisfy a systematic full sampling of the k-space center, a small subset (< 5%) of circles is positioned in rosette pattern in each ECCENTRIC encoding planes. (D), 3D k-space sampling is achieved by a stack of ECCENTRIC encoding planes with variable $k_{x,y}^{max}$ to realize an ellipsoid coverage. (E) Diagram of the FID-ECCENTRIC 3D sequence. First, a 4-pulse WET water suppression technique is used, preceding the Shinnar–Le Roux optimized excitation pulse. After the excitation, the Cartesian encoding is performed along the z-axis, simultaneously to the gradient ramp along the x- and y-axes to reach the desired k-space off-center position and velocity. Finally, a sinusoidal gradient waveform is applied along the x- and y-axis during acquisition to produce the circular trajectory.

To accomplish these aims, we developed ECCENTRIC method (ECcentric Circle ENcoding TRajectorIes for Compressed sensing), which provides several benefits, including: 1) higher acceleration of MRSI acquisition, 2) improved pseudo-random sampling for compressed sense with non-Cartesian trajectory, 3) flexible and optimal sampling of the 4D (k,t) space and 4) reduced demand on the gradient system. Circular trajectories, including rosettes, concentric, and wave circles, provide several advantages over spiral and echo-planar trajectories in MRSI and MRI [23, 24]. By design, ECCENTRIC’s circular trajectories have reduced diameter produced by readout gradient wave-forms that: 1) do not need rewinding which eliminates dead-time and the associated loss in SNR per unit time, 2) permit high matrix sizes with limited gradient amplitude, 3) have constant and moderate gradient slew-rate that is not demanding for patients nor gradient hardware, hence minimizing nerve stimulation and artifacts caused by eddy currents, gradient warming, field drift and mechanical resonances. Moreover, the implementation of CS acceleration [25, 26] relies on two prerequisites. The first is that the signal or image exhibits sparsity in a known transform domain [27, 28]. The second is that the data are randomly undersampled, which can be achieved by random undersampling of the k-space in MRI applications. To enable the random sparse undersampling necessary for Compressed Sensing (CS), we utilized a novel approach where successive circular trajectories are randomly positioned in k-space, rather than using regular patterns such as rosette, concentric, or wave circles (Fig.1). The flexibility of the ECCENTRIC design, which includes a random distribution of circle centers and the ability to choose circle radii freely provides complete flexibility in selecting the matrix size, field-of-view (FoV), and spectral bandwidth. This design also ensures that the gradient hardware’s slew-rate and amplitude limitations are not exceeded, while simultaneously minimizing acquisition time with CS acceleration. Furthermore, it enables better fulfillment of the random undersampling requirement for compressed sensing when compared to echo-planar [29–34], spiral [35] and radial [15] trajectories used in previous accelerated MRSI studies. To reconstruct ECCENTRIC data into image-frequency space, a low-rank (LR) model constrained with Total-Generalized-Variation (TGV) was used: CS-SENSE-LR [36–38]. In addition to the spatial regularization which is required for CS acceleration, the CS-SENSE-LR reconstruction model integrates partial-separability (or low-rank) to further improve the SNR of UHF MRSI. The performance of the new acquisition-reconstruction scheme was first investigated by simulations and in structural-metabolic phantoms, and subsequently evaluated *in vivo* in healthy subjects and glioma patients.

## II. THEORY

### A. ECCENTRIC sampling

ECCENTRIC is a SSE sampling strategy following a random pattern made of off-center circles as illustrated in Figure 1. The circle centers’ polar coordinates $\left(r_{c},\phi_{c}\right)$ are chosen randomly with a uniform probability within the ranges $r_{c}\in \left[0,max\left(k_{x,y}^{max}-R,R\right)\right]$ and $\phi_{c}\in \left[0,2\pi [\right.$. Here, R represents the circle radius, $k_{x,y}^{max}$ is the largest in-plane k-space coordinate (assuming the same spatial resolution along all axial plane directions). The majority of circles are placed randomly as shown with two successive circles (c and c+1) in the sketch A, but with the constraint to avoid significant overlap between circles ( Fig.1B): the distance between the centers of each circle, Δ, must be larger or equal to the Nyquist distance (the inverse of the FoV size). There is partial overlap between circles when Δ<2R, but this redundant sampling occurs primarily in the center of k-space, which allows to optimize and increase the SNR. In addition to the random pattern, a small subset of circles (< 5% of the total number) positioned in rosette fashion is acquired in the center of k-space (Fig.1C). This ensures complete sampling of the center of k-space, which is beneficial for SNR and reconstruction performance [27, 28] with negligible effect on acquisition time. The homogeneous random distribution of circle polar coordinates results intrinsically in a pseudo-random k-space sampling with density following 1/∥k∥ outside of the rosette sampled central region.

To extend ECCENTRIC to 3D k-space sampling, a stack of ECCENTRIC is employed with circles randomly placed in the $k_{x}-k_{y}$ planes, while $k_{z}$ is encoded using Cartesian phase-encoding (Fig.1D). The 3D k-space can be covered using spherical or ellipsoid coverage, where the in-plane k-space boundary is defined as $k_{x,y}^{max}=\frac{M}{2FoV}\sqrt{1-{\left(k_{z}/k_{z}^{max}\right)}^{2}}$, with FoV representing the FoV size and M the spatial resolution (matrix, refer to Fig. 1). To achieve complete sampling in a single $k_{x}-k_{y}$ plane, the number of eccentric circles needed can be derived similar to rosette encoding that requires $\frac{\pi M}{2}$ circles. To maintain the same number of sampling points, the total number of eccentric circles required for complete sampling of a single stack is $\frac{\pi Mk_{x,y}^{max}}{2R}$. To achieve circle encoding with off-center position $\left(r_{c},\phi_{c}\right)$, a brief gradient ramp is used to gain an initial momentum $\left(k_{x},k_{y}\right)$ position and the necessary velocity. This process is done at the same time as the slab excitation rewinder overlapped by z-phase encoding and does not increase the echo time, as shown in Fig.1E. To implement CS acceleration, the total number of ECCENTRIC circles $N_{c}$ is reduced uniformly across the stacks by a factor of AF.

**FIG. 2:**
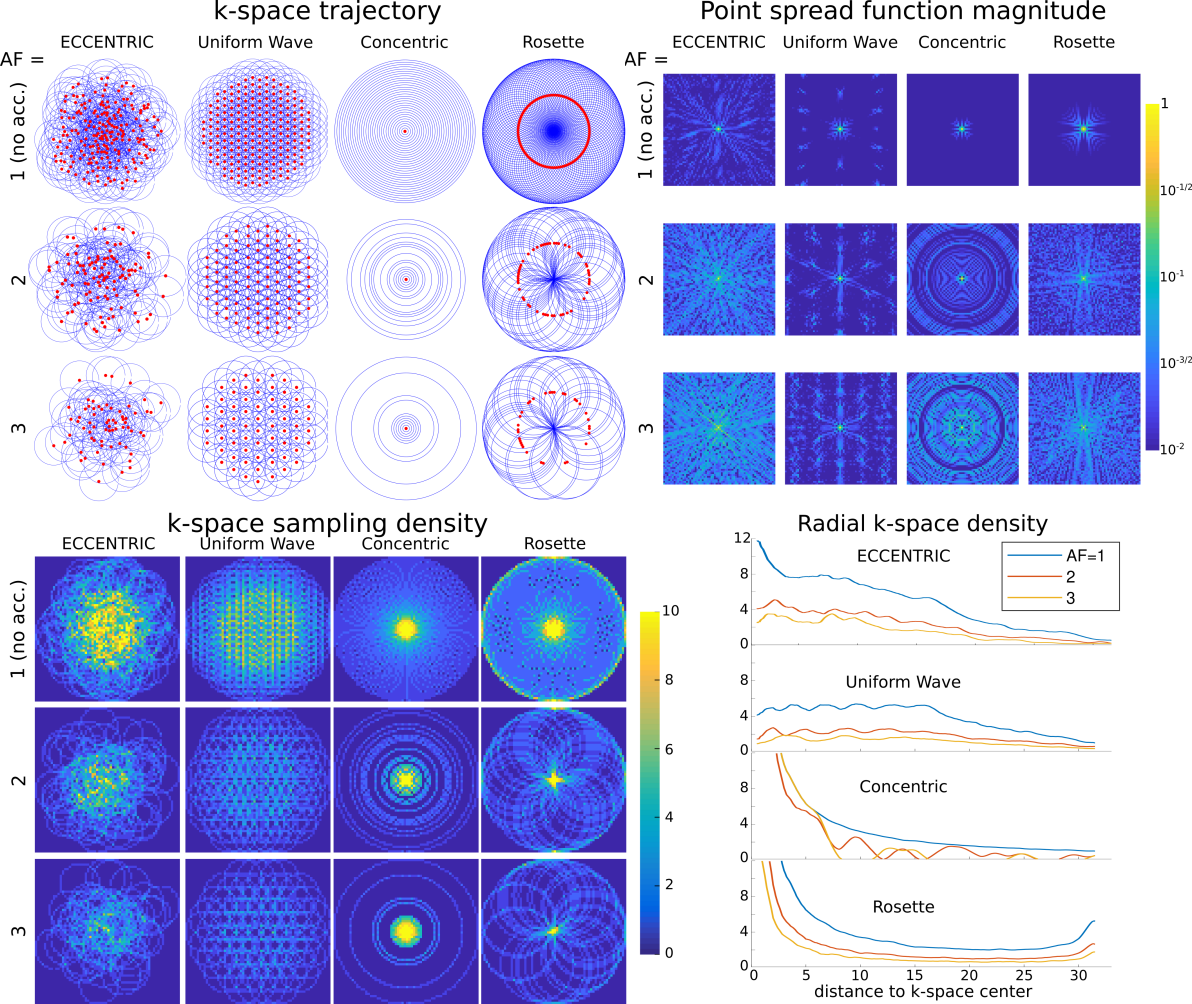
Top left, k-space trajectories for ECCENTRIC, Uniform Wave, concentric circles and rosette trajectories for a 64 × 64 encoding matrix. Red dots indicate the circle center positions. The acceleration factors AF=1, 2, 3 correspond to ECCENTRIC and Uniform Wave trajectories with 202, 101 and 67 circles respectively; 31, 16 and 11 concentric circles; 101, 51 and 34 Rosette circles. Top right, the point spread function (PSF) calculated for each trajectory and acceleration on a log-scale highlight the presence of incoherent and coherent aliasing patterns. Bottom, the sampling density for the same trajectories and AFs, represented in the 2D k-space (left) and along a radial projection (right).

In Fig.2 a comparison is made between ECCENTRIC, Uniform Wave trajectory (similar to Wave-CAIPI pattern [39]), concentric circles and rosette sampling. The trajectory and sampling density in the k-space for each pattern and acceleration factor highlight the differences in sampling distribution. The density distribution of ECCENTRIC is between the density profile of rosette/concentric circles that have a high density singularity at the k-space center and the Uniform Wave which has a flatter profile up to the periphery of the k-space. While rosette and concentric circle trajectories provide a favorable sampling density at the center of k-space, they require temporal interleaving that increase the acquisition time and create sidebands and spectral artefacts which are detrimental to the metabolite signal. Uniform Wave has less desirable k-space density, but does not need temporal interleaving similar to ECCENTRIC. Hence, ECCENTRIC provides a good compromise between the desired high density in the center of k-space to improve SNR and avoiding temporal interleaving.

ECCENTRIC is inherently suited for random undersampling required for good CS performance [27], as demonstrated by comparing (Fig.2) the point spread functions (PSF) of ECCENTRIC, rosette, concentric and Uniform Wave. The PSF was computed on a 64 × 64 matrix and obtained from a single point source [40] that was encoded in k-space and then reconstructed with Non-uniform Fourier transform with Voronoi’s partition density compensation [41]. The PSF reflects the interference between voxels in image space resulting from undersampling. A PSF that shows pseudo-random incoherent pattern is more suited for CS acceleration. Simulations reveal an incoherent pattern for PSF of ECCENTRIC due to the pseudo random k-space sampling, and the PSF pattern spreads with the increasing acceleration factor but conserve the pseudo-random behavior with undersampling (Fig.2). In comparison, Uniform Wave, concentric circles, and rosette trajectories have more compact PSFs for fully sampled acquisitions, but their PSFs exhibit coherent patterns when undersampling is applied which is less favorable for CS acceleration.

### B. Reconstruction of ECCENTRIC data

The CS-SENSE-LR model reconstruction [37, 38] was used to reconstruct 4D ECCENTRIC (k,t) data into image-frequency spacee. Here, we adapted this reconstruction to the case of ECCENTRIC data by incorporating the non-uniform Fourier transform (NUFT).

Defining the discrete MRSI data in image space to be ρ as an $N_{r}$ by T array (with $N_{r}$ the number of spatial points and T the number of sampling time points), the low-rank hypothesis on the magnetization assumes that the MRSI data can be separated into a small number of spatial and temporal components :

(1) ρ=UV

where U is a $N_{r}$ by K array and V a K by T array, with K the rank of the low-rank model. These components are retrieved by CS-SENSE-LR reconstruction solving the inverse problem

(2) $$arg\;\underset{U,V,L}{min}\;∥𝒲(s-ℱ𝒞ℬ(UV+L)){∥}_{2}^{2}\;\;+\lambda \sum_{c=1}^{K}\;\;{TGV}^{2}\left\{U_{c}\right\}.$$,

where s the measured data, ℱ the NUFT encoding operator, 𝒞 the coil sensitivity operator, ℬ the $B_{0}$ frequency shift operator and L represents the lipid signal ($N_{r}$ by T array) located at the skull that is reconstructed simultaneously with brain metabolite U and V but on a separate spatial support. 𝒲, is a weighting operator of a Hamming window shape, and decreasing with the distance to the center of the k-space [37, 42]. TGV^2^ is the total generalized variation cost function with λ the regularization parameter [43]. The NUFT encoding operator of ECCENTRIC ℱ is a discrete non-uniform Fourier transform of type 1:

(3) $${\left(ℱ\rho \right)}_{j,t}=\sum_{i}{\text{e}}^{2\pi ik_{j}\cdot r_{i}}\rho_{i,t}$$

with $r_{i}$ are the uniform image space coordinates and $k_{j}\;k$-space sampling-point coordinates located on the 3D-ECCENTRIC circles. The contamination of U and V by skull-lipid signal can be prevented by filtering of the gradient descent during the reconstruction (eq.2). Lipid signal is removed from each step of the gradient descent by applying the operator (1-P) with P the lipid subspace projection computed from the estimated lipid signal at the skull L [36]. To remove residual water signal remaining in measured data, HSVD method for water removal is applied to the raw data [44]. At last, the first order phase that is inherent to the acquisition delay in FID-MRSI type acquisition, are removed by predicting the first missing points with an autoregressive model [45].

## III. METHODS

### A. ECCENTRIC sequence and acquisition parameters

A ^1^H-FID-MRSI [6, 8] acquisition was implemented with 3D spherical stack-of-ECCENTRIC sampling on a 7T scanner (MAGNETOM Terra, Siemens Healthcare, Erlangen, Germany) running VE12U SP1 software and equipped with a NOVA head coil (32Rx/1Tx). The echo-time (TE) was set to the minimum possible: 0.9 ms with a 27 degree excitation flip-angle (FA) and 275 ms repetition-time (TR). A slab selective excitation was performed with a Shinnar-LeRoux optimized pulse [37, 46] with 6.5 kHz bandwidth and was preceded by four-pulses WET water suppression scheme [37, 47] (Fig.1E). The FoV was 220 × 220 × 105 mm^3^ (A-P/R-L/H-F) with 85 mm-thick excited slab. A voxel size of 3.4 × 3.4 × 3.4 mm^3^ (40.5μl) was realized with a 64 × 64 × 31 matrix. The ECCENTRIC circles radius R was set to $1/8\frac{n}{FOV}$ which corresponds which corresponds to a diameter that encompasses a quarter of the width of the k-space, with n being the square matrix size. With the chosen radius R, each ECCENTRIC circle sampled 51 points in k-space, enabling a spectral bandwidth of 2280 Hz without the need for temporal interleaving. The FID was sampled with 500 time-points, which resulted in a total FID duration of 220 ms. To obtain the fully sampled (AF=1) spherical 3D Stack-of-ECCENTRIC with these parameters, a total number of $N_{c}=4072$ circles is required, which corresponds to 18 min 40 sec acquisition time (TA). For accelerated acquisitions, we decreased the number of circles to $N_{c}/AF$, with TA being shortened proportionally. For instance, with the same encoding parameters, AF=2 needs $N_{c}=2036$ in 9 min 20 sec, AF=3 needs $N_{c}=1357$ in 6 min 16 sec, AF=4 needs $N_{c}=1018$ in 4 min 40 sec, and so on. A very fast water reference data needed for coil combination, $B_{1}$ and $B_{0}$ field correction (𝒞ℬ operators in Eq.2), and image intensity normalization for comparable range of metabolite concentration values across subjects (in institutional units, I.U.) was acquired by turning off water suppression and using the same FoV, FA slab excitation, TR, FID duration and spectral bandwidth, with Rosette sampling at lower resolution (23 × 23 × 19) in 1 min 16 sec. The 3D-ECCENTRIC ^1^H-FID-MRSI data were processed by removing any residual water and then reconstructed using a TGV regularized model that also includes simultaneous suppression of scalp lipid signal (Eq. 2).

In healthy subjects and patients shimming of the 85 mm thick whole-brain slab was performed using the manufacturer methods that adjusted the shim currents over thirteen spherical harmonics coils: three 1st order, five 2nd order, and four 3rd order. The global linewidth of the water over the entire 85 mm slab was between 25–42 Hz across all subjects. In the majority of the subjects the global water linewidth was between 30–35 Hz. Adjustment of the $B_{1}$+ transmit and water suppression was subsequently performed with manufacturer methods. The entire adjustment procedure took between 1–2 min for every subject.

### B. ECCENTRIC metabolite maps acquisition on healthy volunteers

Twenty healthy volunteers (11 female, 9 male, 22–42 years, listed in Table I) were scanned at Athinoula A. Martinos Center For Biomedical Imaging for this study. In four volunteers, the 3D-ECCENTRIC ^1^H-FID-MRSI sequence described above was acquired with voxel size of 3.4 mm isotropic at AF= 1,2,3 and 4 successively. In two healthy subjects the performance of 3D-ECCENTRIC ^1^H-FID-MRSI was tested at higher resolution with voxel size of 2.5 mm isotropic (matrix 88 × 88 × 43, AF=4, TA=10 min 26 sec) and compared to 3.4 mm isotropic (matrix 64 × 64 × 31, AF= 2 (TA= 9 min 20 sec). In the remaining volunteers, the 3D-ECCENTRIC ^1^H-FID-MRSI was acquired only with 3.4 mm isotropic and AF=2 (TA= 9 min 20 sec)

All volunteers were scanned with a $T_{1}$-weighted anatomical MP2RAGE sequence [48] (1 mm isotropic, 4300 ms TR, 840 ms & 2370 ms TI) for positioning of the MRSI FOV and for the generation of skull-masks that are needed for the lipid suppression during the reconstruction and to exclude voxels located outside the head volume. The regularization parameter used in the reconstruction was adjusted to $\lambda =3\times 10^{-4}$ by gradually increasing it from a low value until the noise-like artifacts in the metabolite maps disappeared [38, 43]. The reconstruction rank, K, was determined qualitatively as the minimum number of components that contain some signal distinguishable from noise. For the 3D ECCENTRIC reconstruction, K was specifically set to 40.

### C. Glioma patient metabolite imaging with 3D-ECCENTRIC ^1^H-FID-MRSI

Twenty patients (11 male, 9 female, 28–66 years) were imaged at Athinoula A. Martinos Center For Biomedical Imaging with 3D-ECCENTRIC ^1^H-FID-MRSI to assess the performance for mapping metabolic profile in disease conditions. Seventeen patients had biopsy confirmed diagnosis: 1) nine patients were diagnosed with astrocytoma (WHO 2–4), 2) seven patients were diagnosed with oligodendroglioma (WHO 2–3), and 3) one patient was diagnosed with glioblastoma (WHO 4). IDH1(R132H) mutation was confirmed in sixteen patients, and 1p/19q codeletion was confirmed in three patients. In all patients the 3D-ECCENTRIC ^1^H-FID-MRSI was acquired with 3.4 mm isotropic and AF=2(TA= 9 min 20 sec). In addition, 2.5 mm isotropic (AF=4, TA= 10 min 26 sec) protocol was acquired in two patients for comparison with the 3.4 mm isotropic protocol.

### D. Quantitative and Statistical Analysis of Metabolic Imaging

After reconstructing the ECCENTRIC MRSI data, the LCModel software [49] was used to fit the data and quantify the metabolites. A metabolite basis obtained from quantum mechanics simulations in GAMMA [37] was utilized for quantification, consisting of twenty-one metabolites: phosphorylcholine (PCh), glycerophosphorylcholine (GPC), creatine (Cr), phosphocreatine (PCr), gamma-aminobutyric acid (GABA), glutamate (Glu), glutamine (Gln), glycine (Gly), glutathione (GSH), myo-inositol (Ins), N-acetylaspartate (NAA), N-acetyl aspartylglutamate (NAAG), scyllo-inositol (Sci), lactate (Lac), threonine (Thr), beta-glucose (bGlu), alanine (Ala), aspartate (Asp), ascorbate (Asc), serine (Ser), taurine (Tau). Phosphorylcholine and glycerophosphorylcholine were combined into total choline-containing compounds (Cho), while creatine and phosphocreatine were combined into total creatine (tCr). For the analysis of glioma patients data, 2-hydroxyglutarate (2HG) was also included in the basis. Concentration maps were then generated for the metabolites included in the simulated basis.

The reconstructed water reference signal was used as quantification reference by LCModel and the resulting concentration estimates were expressed in institutional units (I.U.). This allowed for comparisons of metabolite levels across both subjects and different metabolites. The ultra-short TE used in the ECCENTRIC MRSI data acquisition meant that $T_{2}$ relaxation correction was unnecessary for both metabolite and water signals. The results of the LCModel fitting for each voxel were then used to generate spatial maps of the concentration of each metabolite. To assess the quality of the MRSI data and the goodness of fit, quality control maps of Cramer-Rao lower bounds (CRLB), line-width (FWHM), and signal-to-noise ratio (SNR) were generated. MP2RAGE images of healthy volunteers were segmented into gray-matter, white-matter and CSF with Freesurfer version 6.0.0 software [50], and cerebral lobes were identified using a standard atlas [38]. Tumor regions of interest (T-ROIs) and healthy contralateral regions of interest (CL-ROIs) were segmented using ITK-SNAP on FLAIR imaging in glioma patients. The metabolic tumor contrast-to-noise ratio $\left(CNR=\frac{\mu_{T}-\mu_{H}}{\sigma_{H}}\right)$ was computed for all metabolites, where $\mu_{T,H}$ represent the mean of the metabolite concentrations in tumor and healthy appearing brain, while $\sigma_{H}$ represents the standard deviation of the metabolite concentration in the healthy appearing brain. To compare the metabolite concentrations (IU) in the tumor and tumor CNR across groups, an ANOVA was carried out, followed by post-hoc multiple comparisons to identify group means with significant differences. We performed a Wilcoxon signed-rank test on the metabolite tumor concentrations and CNR with multiple comparison correction to compare treatment-naïve mutant IDH1 patients with the remaining patients (treated mutant IDH1 and wild-type IDH1/2 combined). Furthermore, we conducted a Wilcoxon signed-rank test including all patient data to determine which metabolite had median CNR values significantly different from zero.

## IV. RESULTS

### A. ECCENTRIC metabolic imaging in healthy volunteers

Guided by simulations, phantom and water imaging shown in Fig.2 and Supplementary Fig. S2 and S1, next we investigated ECCENTRIC performance for whole-brain metabolic imaging in healthy volunteers. 3D-ECCENTRIC ^1^H-FID-MRSI was performed with a voxel size of 3.4 mm isotropic using acceleration factors between 1 to 4. We sought to find whether a metabolic imaging protocol that provides 3 mm isotropic whole-brain coverage in under 5 minutes (AF=4) is feasible by ECCENTRIC for routine clinical exams. Such performance is similar to other advanced MR imaging methods such as CEST and perfusion imaging.

**FIG. 3:**
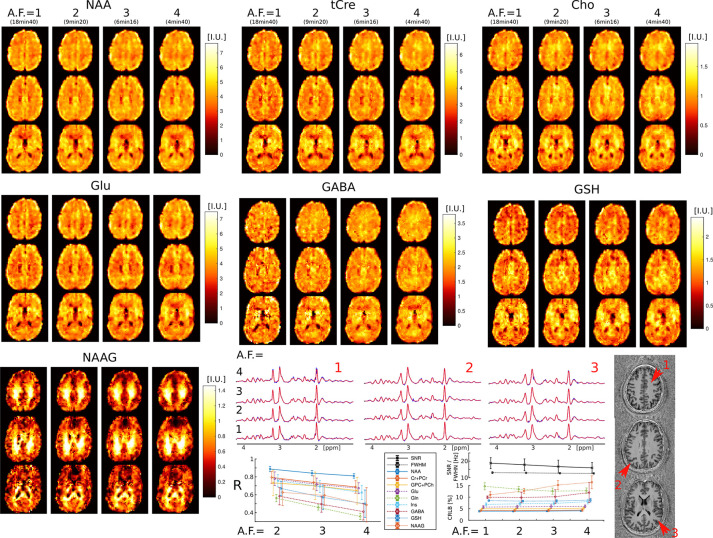
3D-ECCENTRIC ^1^H-FID-MRSI metabolic images of human brain in healthy volunteer with 3.4 mm isotropic voxel size and 1–4 CS acceleration factors. Top, metabolite maps of seven relevant brain metabolites (NAA, tCre, Cho, Glu, GABA, GSH, and NAAG) are shown for all acceleration factors (AF). Spectra from three brain locations indicated by red arrows on the anatomical image. At the bottom, the left plot displays the correlation coefficients between accelerated images (AF=2,3,4) and fully sampled images (AF=1), while the right plots show the LCModel quantification error (CRLB), linewidth (FWHM), and SNR.

Examples of metabolic images for seven metabolites obtained with retrospective AF=1-4 are shown in Fig.3. Very similar structural details and tissue contrast of metabolic images are obtained for all accelerations compared to the fully sampled data. This is visible also by inspecting spectra that show the same metabolic profile across acceleration factors (AF=1-4). The CS accelerations (AF=2,3,4) were achieved by retrospectively undersampling the fully acquired data (AF=1). The purpose of this was to solely focus the analysis on the effects of CS acceleration, while also avoiding any image differences that could be caused by patient motion during acquisitions with different accelerations.

Visual inspection of metabolic images reveal that: 1) tCre, Glu and GABA have larger signal in gray matter than white matter, 2) NAA has more signal in gray than white matter, but with lower gray-white matter contrast compared to tCre, Glu and GABA, 3) Cho has higher signal in frontal white matter than gray matter, 4) NAAG has the largest contrast from all metabolites, with much larger signal in white matter compared to gray matter. Metabolic images obtained with AF=1 and AF=2 are largely identical. Minor blurring of fine structural details starts to become noticeable for AF≥3, however adequate delineation of gray-white matter folding is maintained up to AF=4.

Quantitative image analysis shows that the correlation between the accelerated and fully sampled metabolic images is high (R>0.7) for metabolites that have a high SNR such as NAA, Cho, tCr, Glu and Ins, while metabolites of lower SNR such as Gln, GABA, GSH, and NAAG exhibit lower correlations (R=0.4-0.7). The error (CRLB) of metabolite quantification is below 20% for all nine metabolites, and it does not degrades with the acceleration factor except for NAAG and GABA, albeit it does not exceeds the 20% limit up to AF=4. The SNR shows only a minor decrease between AF=1 (SNR=17) and AF=4 (SNR=15), while the linewidth does not change. More detailed analysis is presented in the Table II and the following section.

### B. ECCENTRIC metabolic imaging in glioma patients

Performance of the 3.4mm isotropic 3D-ECCENTRIC ^1^H-FID-MRSI was investigated in twenty patients with glioma brain tumors. Table I lists pathology, molecular diagnosis, treatment history and demographics of the patients.

**FIG. 4:**
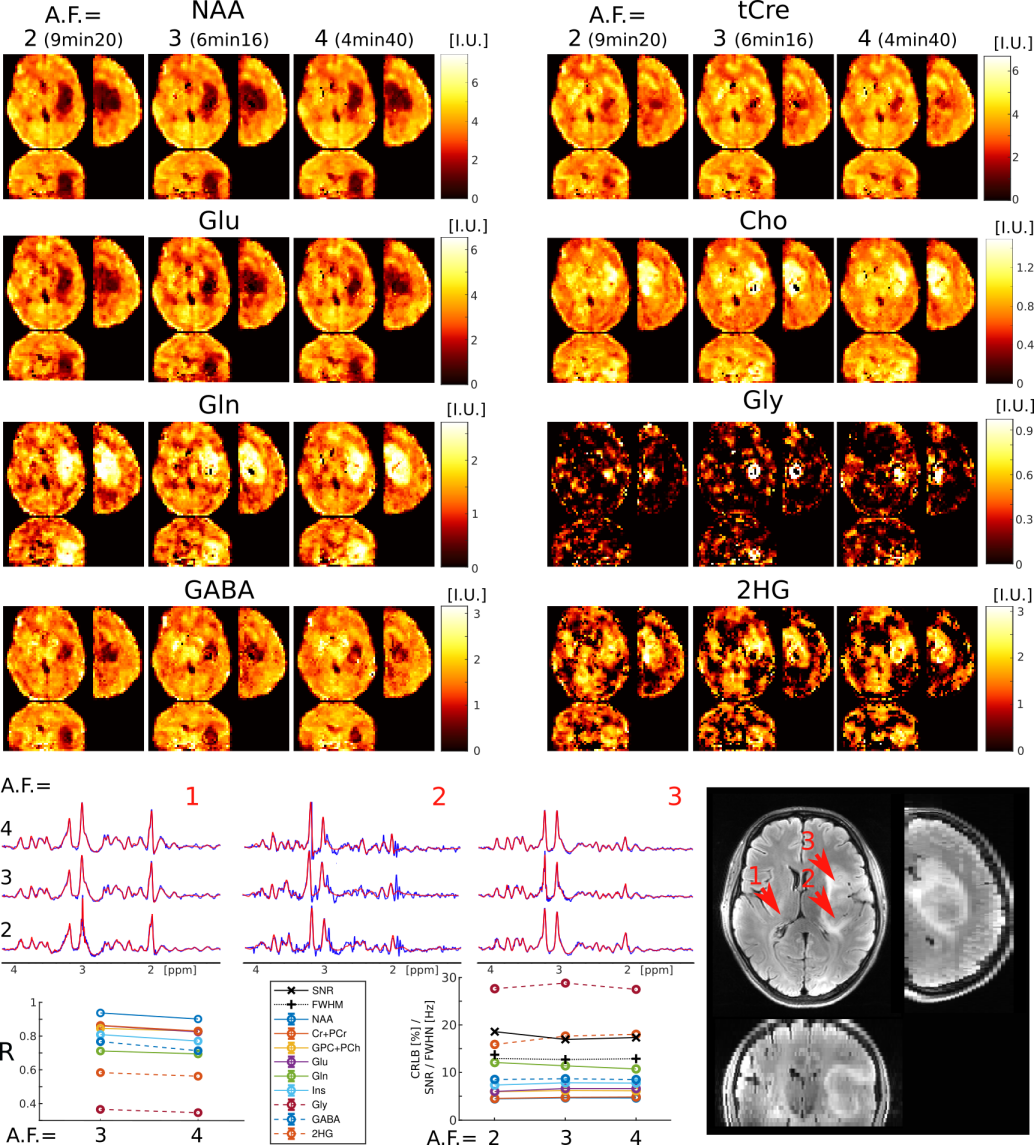
ECCENTRIC metabolic imaging in a WHO-3 Astrocytoma patient with IDH1(R132H) mutation (Patient #2 in Table I). The performance of 3D-ECCENTRIC ^1^H-FID-MRSI, acquired at 3.4 mm isotropic resolution with acceleration factors (AF) of 2, and retrospectively accelerated to 3 and 4, was compared. Top, metabolic images of eight metabolites for all accelerations. Bottom, spectra from three brain locations indicated by red arrows on the FLAIR image, correlation coefficient (R) between AF=2 and AF=3,4, metabolite quantification error estimate (CRLB), linewidth (FWHM), and signal-to-noise ratio (SNR) across AF.

The results of retrospective acceleration on the spatial mapping of metabolite profiles in tumor and the healthy appearing brain are presented in Fig.4. Metabolic images from eight metabolites acquired with AF=2 and retrospectively accelerated to AF=3 and 4, are shown. Fully sampled data (AF=1) was not acquired in patients due to its long acquisition time (18min:40s). We demonstrated in both healthy subjects and phantoms that mild undersampling (AF=2) yields metabolic maps that are practically indistinguishable from those fully sampled (AF=1). Hence, in patients we used AF=2 data as ground truth to estimate the performance of higher accelerations (AF=3,4).

Fig.4 shows metabolic images of six important metabolites present in healthy brain, and additionally two metabolites that are specifically present in glioma tumors, such as 2HG associated with IDH1,2 mutations and Gly.

Patient metabolic imaging shows similar performance for different accelerations, similar to healthy volunteers and phantoms measurements. The tumor can be visualized with high contrast-to-noise by all metabolites, in particular NAA, Cho, Glu, Gln, Gly and 2HG. However, the spatial extent and pattern of metabolic abnormality is different across the eight metabolites, which is suggestive of tumor heterogeneity as it is typical with glioma. Examples of spectra from voxels inside and outside the tumor clearly show different metabolic profiles, which are consistently reproduced by all the acceleration factors. Seven metabolite maps (NAA, tCre, Cho, Glu, Gln, GABA, Ins) exhibit high correlation (R>0.7) between ground truth AF=2 and the higher accelerations (AF=3,4). Two metabolites exhibit medium correlation, 2HG (R~0.6) and Gly (R~0.4), which is driven by the low background outside the tumors. However, no noticeable degradation of the correlation coefficients is observed for any metabolite with increasing acceleration, while slight blurriness consecutive to CS acceleration appears for AF=4. The spectral quality metrics CRLB, FWHM, and SNR are also stable across acceleration factors.

**FIG. 5:**
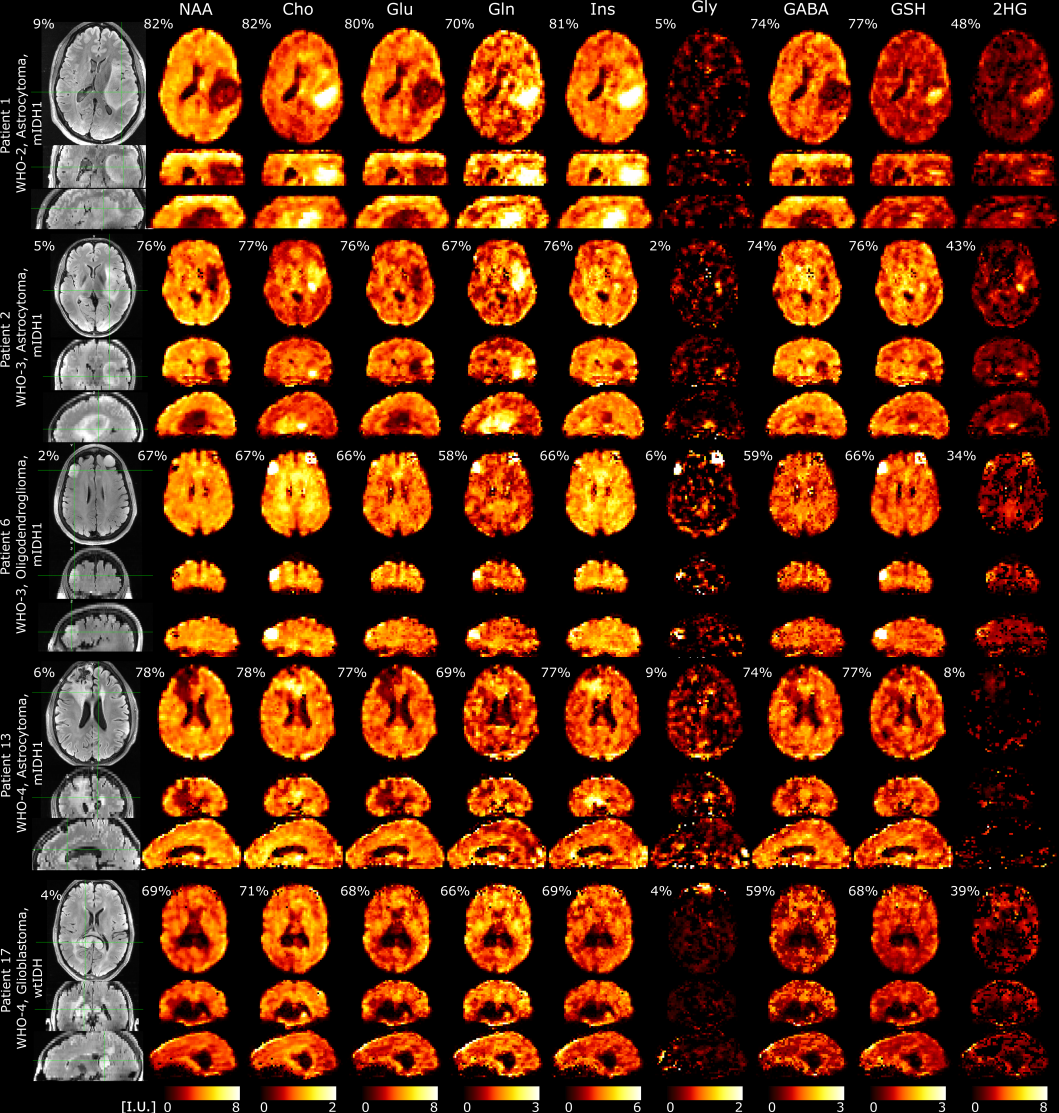
Metabolic imaging in glioma patients acquired with 3D-ECCENTRIC ^1^H-FID-MRSI at 3.4 mm isotropic resolution in 9min:20s accelerated by CS (AF=2). The top four patients have mutant IDH1(R132H) glioma: WHO-2 Astrocytoma, WHO-3 Astrocytoma, WHO-3 Oligodendroglioma, and WHO-4 Astrocytoma (Patients #1, #2, #6, #13 in Table I). The bottom fifth patient has a necrotic wild-type IDH1/2 WHO-4 Glioblastoma (Patient #17 in Table I). Intensity scale (I.U., institutional units) for each metabolite is the same across all patients. For each metabolite, we indicate the percentage of voxels inside the brain and FoV that meet the criteria of acceptable quality (CRLB < 20%, FWHM < 0.07ppm, SNR > 5). Percentage on FLAIR indicate the ratio of tumor volume to the total brain volume.

Examples of metabolic images obtained with 3D-ECCENTRIC ^1^H-FID-MRSI at 3.4 mm isotropic resolution and AF=2 in 9min:20s from five patients, including four mutant IDH1(R132H) and one wild-type IDH1,2 glioma, are shown in Fig.5. Images of nine metabolites are shown in comparison to the FLAIR image.

In the the four mutant IDH1 glioma patients five metabolites exhibit positive contrast for tumor (Cho, Gln, Ins, Gly and 2HG), three metabolites have negative contrast for the tumor (NAA, Glu and GABA), and one metabolite (GSH) shows both positive and negative contrast. In the WHO-4 high grade wild-type IDH1/2 glioblastoma patient all metabolites exhibit negative contrast due to tumor necrosis, which is corroborated by lack of contrast enhancement and perfusion imaging. The different spatial extent and pattern of metabolic abnormality revealed by each metabolite highlights the heterogeneity of glioma metabolism. This emphasizes the complementarity and value of all metabolites for glioma imaging. These data demonstrate that an extended metabolic profile can be imaged at 7T with high quality at high spatial resolution in a clinically acceptable time in glioma patients.

### C. Quantitative analysis of ECCENTRIC metabolic imaging

**FIG. 6:**
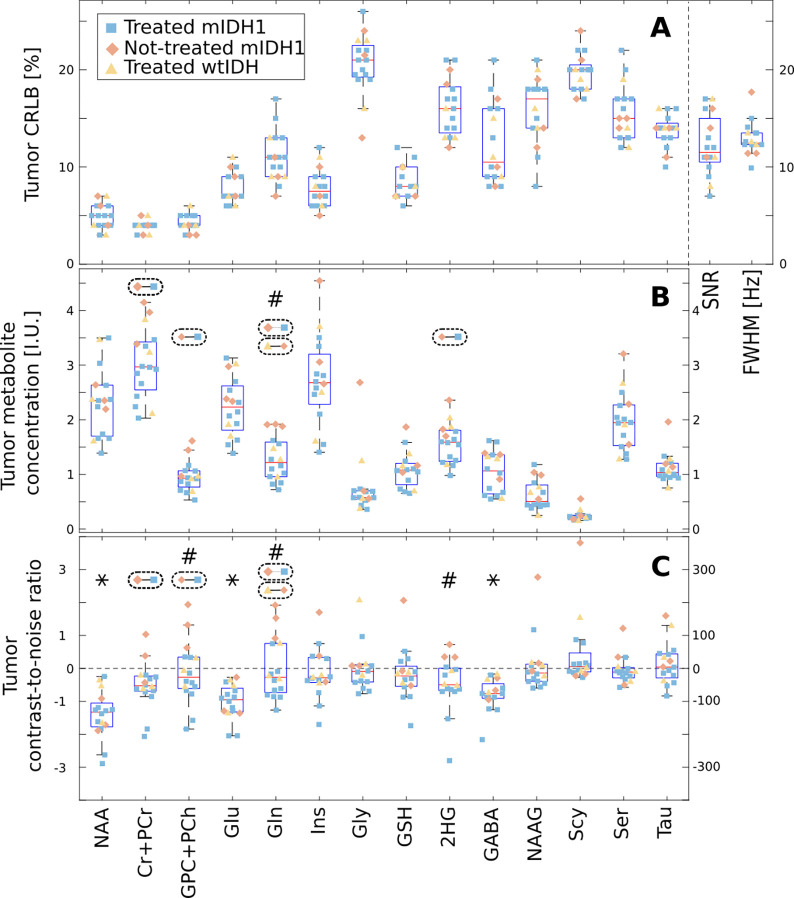
Group analysis of the metabolites within the tumor region of interest (ROI) and normal appearing brain in glioma patients. The shape and color of each point represent the patient group, and the box plots display the median, 25th, and 75th percentiles for all patients. The **A** plot shows the mean values of Cramer-Rao lower bound (CRLB), signal-to-noise ratio (SNR), and linewidth (FWHM) within the tumor ROI. The **B** plot shows the mean metabolite concentration within the tumor ROI in IU. The **C** plot shows the contrast-to-noise ratio (CNR) between the tumor ROI and normal appearing brain. ANOVA with post-hoc multiple comparisons across the three groups was performed for each metabolite and the dashed ellipses with two linked points indicate groups that have a significant difference. In **B and C**, metabolites marked with a # indicate a significant difference, as determined by a Wilcoxon sign-rank test corrected for multiple comparisons, in the tumor concentration or in the contrast-to-noise ratio (CNR) between treatment-na”ive mutant IDH1(R132H) patients and other patients. In **C**, the * denotes metabolites for which the median CNR value computed over all patients was significantly different from zero (corrected Wilcoxon signed-rank test). In all plots only the ROI voxels with CRLB < 30% were included in the analysis.

Detailed quantitative analysis including regional metabolite concentrations, quantification error estimates, and spectral quality metrics is presented in Table II and Fig.6 for healthy volunteers and glioma patients, respectively.

Table II presents the regional concentrations of nine metabolites quantified with respect to the reference water signal and expressed in IU, calculated in both the gray and white matter of the five major brain lobes across healthy volunteers. Our results indicate that: 1) six metabolites have higher concentrations in gray-matter compared to white matter across the brain (GM/WM = 1.19 tCre, 1.12 NAA, 1.2 Glu, 1.34 Gln, 1.15 GABA, 1.11 GSH); 2) two metabolites have higher concentrations in white-matter compared to gray-matter (GM/WM = 0.96 Cho, 0.47 NAAG); 3) one metabolite has region dependent gray/white-matter ratio (GM/WM = 1.17–0.87 Ins). The largest gray-white matter contrast is exhibited by NAAG due to its specific compartmentalization in white matter.

The quality metrics of MRSI data are also listed in Table II, including the precision of metabolite quantification by the Cramer-Rao lower bounds, the signal-to-noise ratio and spectral linewidth. It can be seen that mean CRLB is below < 20% for all the metabolites across the imaged whole-brain volume. In particular, mean CRLB is below < 6% for the five metabolites with highest SNR (NAA, tCre, Cho, Glu and Ins), between 8%-10% for two metabolites (GABA and GSH) and between 14%-20% for other two metabolites (Glu and NAAG). Across the brain the mean SNR is larger than 20 and the mean linewidth is less than 12 Hz (0.04 ppm).

Stability and reproducibility of metabolite quantification by ECCENTRIC was investigated by test-retest of repeated imaging in four healthy volunteers. Results are shown in Supplementary Fig.S3 and Supplementary Tables S1 and S2 obtained at 3.4 mm isotropic resolution acquired in four consecutive 3D ECCENTRIC scans using AF=1 (18min:40s), AF=2 (9min:20s), AF=3 (6min:16s), and AF=4 (4min:40s). Across all four scans in all four subjects the metabolite images appear visually similar, with low (< 7%) inter-scan coefficient of variation for the concentration of the main metabolites.

Fig.6 presents results of metabolite quantification in the glioma patients. Out of 20 scanned patients (Table I), the MRSI data quality was good and could be analyzed in 19 patients, while the MRSI data in one patient was affected by motion and was excluded from the analysis. The patients with pathology confirmed diagnosis were grouped in three groups based on their IDH mutation status and the treatment history: 1) three newly diagnosed mutant IDH1(R132H) glioma with no prior treatment, 2) eleven mutant IDH1(R132H) glioma with prior treatment, and 3) three wild-type IDH1/2 glioma with prior treatment. Note, that two patients were scanned before any diagnosis and found to have high 2HG levels indicative of mutant IDH1 glioma, which was later confirmed by biopsy. Three patients have a presumptive diagnosis of glioma based on the imaging features, however their diagnosis is not confirmed at the moment by immunohistopathology due to inoperable tumor location and the high risk of biopsy. Hence, these three patients were not included for statistical analysis in any of the three groups.

An extended metabolic profile of fourteen metabolites is quantified and compared in the tumor region-of-interest for the sixteen patients that have biopsy confirmed diagnosis and good data quality. In the top plot (A) of Fig.6 a mean CRLB < 20% is noted for eight metabolites in the tumor, while the mean CRLB is < 27% for the remaining six metabolites. In the tumor the mean SNR of spectra is between 7–17, which is lower than healthy brain due to low tumor NAA, while the mean FWHM is between 10–18 Hz (0.03–0.06 ppm) comparable to healthy brain. In the middle plot (B) of Fig.6 the comparison of metabolite concentration in the tumor indicates that four metabolites have statistically significant different levels between the three patient groups: 1) tCre is higher in treatment-naïve mutant IDH1 compared to treated mutant IDH1 (+1.0 IU, p=0.02), 2) Cho is higher in treatment-naïve mutant IDH1 compared to treated mutant (+0.47 IU, p=0.014 ), 3) Gln is higher in treatment-naïve mutant IDH1 compared to treated mutant and wild-type IDH1/2 (+0.79 IU, p<0.002, +0.64 IU, p=0.03), and 4) 2HG is higher in treatment-naïve mutant IDH1 compared to treated mutant IDH1 (+0.55 IU, p=0.046). In the bottom plot (C) of Fig.6 the tumor contrast-to-noise ratio (CNR) of metabolites across all patients indicates that three metabolites (NAA: −1.3, p<0.001; Glu: −0.96, p<0.001; GABA: −0.77, p<0.001) have statistically significant negative image contrast between tumor and normal appearing brain. In the three groups comparison it can be seen that treatment-naïve mutant IDH1 glioma patients have a CNR for Cho greater (+1.87, p=0.003) than treated mutant IDH1, and that Gln’s CNR is higher in treatment-naïve mutant IDH1 compared to treated mutant IDH1 (+1.38, p=0.03) and wild-type IDH1/2 (+1.95, p<0.001). In general, it can be seen that the overlap between treated mutant IDH1 and wild-type IDH1/2 metabolic profiles is larger than between either of these two groups and the treatment-naïve mutant IDH1 glioma. Standard-of-care has been shown to reduce significantly the levels of 2HG in mutant IDH1 patients [51, 52]. Hence, we conducted a two-group comparison between treatment-naïve mutant IDH1 patients and the remaining patients (treated mutant IDH1 and wild-type IDH1/2 combined). Upon correction for multiple comparisons, the analysis revealed significantly higher Gln concentration in the tumor of treatment-naïve mutant IDH1 patients compared to the other patients (+0.81 IU, p=0.05). Additionally, treatment-naïve mutant IDH1 patients exhibited a significantly higher tumor CNR compared to the other patients for three metabolites: 1) Cho: +1.75, p=0.05; 2) Gln: +1.90, p=0.05; 3) 2HG: +0.96, p=0.05.

### D. Ultra-high resolution metabolic imaging of human brain using 3D-ECCENTRIC ^1^H-FID-MRSI

**FIG. 7:**
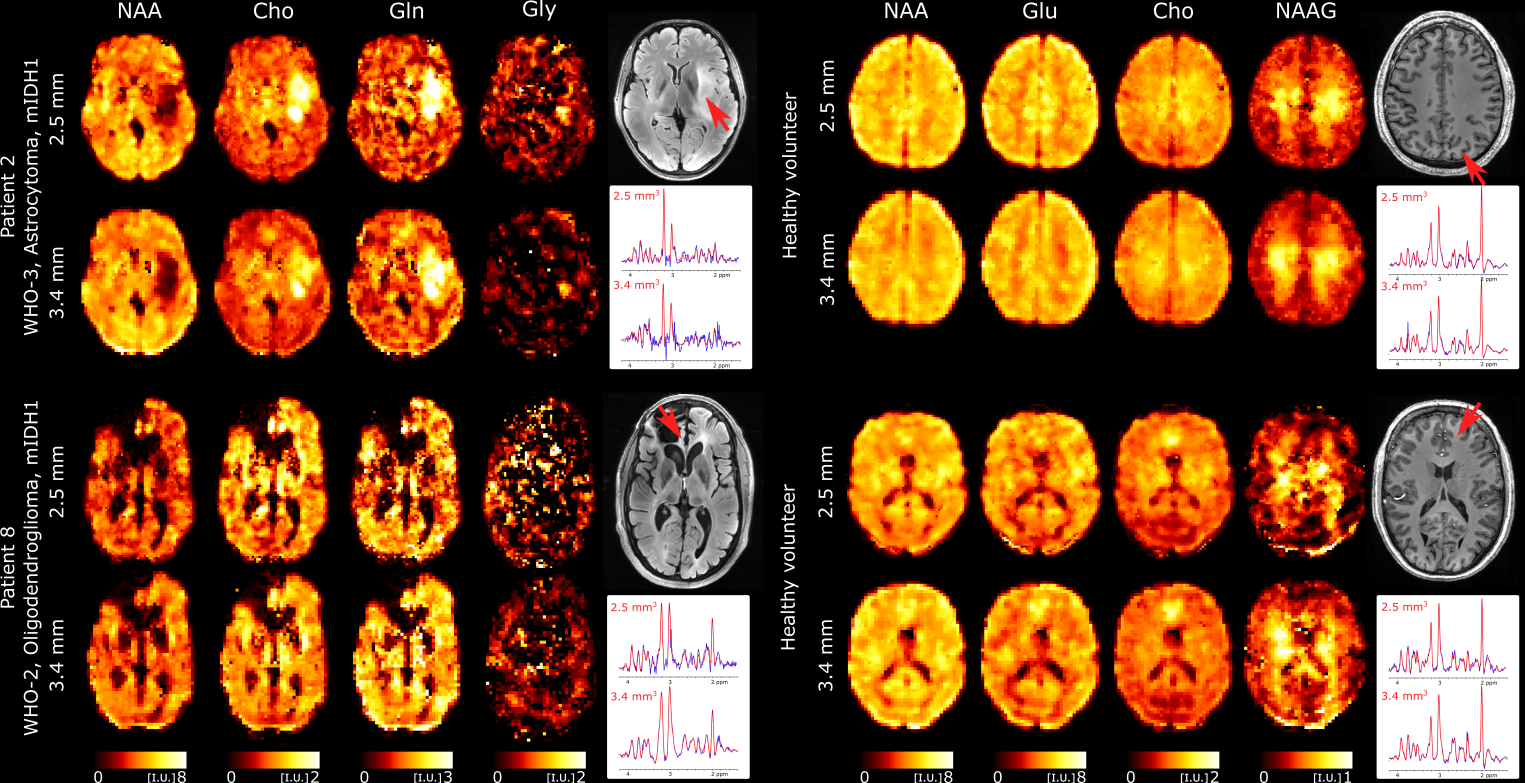
Ultra-high resolution metabolic imaging acquired with 3D-ECCENTRIC ^1^H-FID-MRSI at 2.5mm isotropic voxel size (AF=4, TA=10*min* : 26*s*) in two healthy volunteers and two glioma patients (Patients #2 and #13 in Table I). The ultra-high resolution metabolic imaging is compared to 3D-ECCENTRIC ^1^H-FID-MRSI at the typical voxel size of 3.4mm isotropic (AF=2, TA=9*min* : 20*s*). Two spectra from both spatial resolution and corresponding to the red arrow location are shown. The blue line represents the MRSI data and the red line is the fit performed by LCModel.

We further explored the performance of 3D-ECCENTRIC ^1^H-FID-MRSI for ultra-high resolution metabolic imaging in several healthy volunteers and glioma patients. Based on the high SNR of the 3.4 mm data we expected that smaller voxels at higher resolution will still provide sufficient SNR for metabolite imaging. Fig.7 shows metabolic images obtained at a voxel size of 2.5 mm isotropic using 3D-ECCENTRIC ^1^H-FID-MRSI in two glioma patients and two healthy volunteers.

To achieve a feasible scan time, we used compressed sensing (CS) with AF=4. We demonstrated at the beginning of our work that this AF provides metabolic maps that are similar to those obtained through fully sampled 3D-ECCENTRIC ^1^H-FID-MRSI. The AF=4 acceleration enabled the acquisition of 3D-ECCENTRIC ^1^H-FID-MRSI at 2.5 mm isotropic resolution in 10min:26s. For all the subjects shown in Fig.7 we also acquired the typical 3D-ECCENTRIC ^1^H-FID-MRSI at 3.4 mm with CS AF=2 acceleration (9min:20s). As readily apparent by visual inspection, the metabolic maps at higher spatial resolution provide sharper delineation of the normal brain structure and of the tumor margins. No compromise is visible for signal-to-noise, contrast-to noise or other data quality metric at ultra-high resolution compared to typical resolution. We note that the acquisition time of 3D-ECCENTRIC ^1^H-FID-MRSI at 2.5 mm with AF=4 is only slightly longer (1 min) than at 3.4 mm with AF=2. However, for the same acceleration factor the acquisition time of 3D-ECCENTRIC ^1^H-FID-MRSI at 3.4 mm is 2.2 times faster than with voxel size of 2.5 mm isotropic.

## V. DISCUSSION

In this work we demonstrated that 3D-ECCENTRIC ^1^H-FID-MRSI at 7T can simultaneously image an extended metabolic profile of 10–14 metabolites at high spatial resolution across whole-brain in clinically acceptable time both in patients and healthy controls. In particular, we showed that the acquisition of fast non-Cartesian MRSI can be further accelerated up to four-fold by CS, allowing metabolic imaging at 3.4 mm isotropic resolution in 4min:40s and at 2.5 mm isotropic resolution in 10min:26s, respectively. The CS-SENSE-LR reconstruction method produces metabolic images with an effective voxel size identical to the nominal size [37]. This may provide an advantage compared to other filtered reconstructions [21, 53] which increase the effective voxel volume. ECCENTRIC fully preserves the quality of the metabolite images up to a two-fold acceleration and even when accelerated up to four-fold, the loss of image quality is minor and metabolic images still effectively highlight the laminar structure of the brain and tumor margins. By design ECCENTRIC acquisition and reconstruction preserves the SNR at high accelerations. This is achieved through full sampling of the center of k-space and the use of a low-rank denoising technique.

Here we investigated the performance of 3D-ECCENTRIC ^1^H-FID-MRSI for two applications scenarios: 1) high resolution metabolic imaging (3.4 mm in 4min:40s) for routine clinical applications, and 2) ultra-high metabolic imaging (2.5 mm in 10min:26s) for research applications and more extensive clinical investigations. Both of these protocols, represent a significant advancement for imaging human brain metabolism by non-invasive *in vivo* MRSI.

Results obtained with the clinical 3.4 mm imaging protocol show good delineation of the brain structure and tumor lesions. At 2.5 mm ultra-high resolution there is increased gray-white matter contrast of metabolites due to less partial volume effect that visualise brain structures more clearly than at 3.4 mm. Several metabolites show particularly high contrast between gray and white matter in healthy brain, such as the energy buffer tCre, the neurotransmitter Glu and the dipeptide NAAG. In particular, NAAG is the most abundant dipeptide in the brain, which is selectively localized in several regions [54] where it neuromodulates the glutamatergic synapses, and is implicated in neurodegenerative diseases, schizophrenia, stroke, epilepsy, traumatic brain injury and pain [55]. 3D-ECCENTRIC ^1^H-FID-MRSI provides high quality images of the NAAG brain distribution showing high concentration in parietal white matter. In addition, good quality metabolic images are obtained for some of the most important but challenging metabolites such as GABA, Gln, and GSH. The combination of improved linewidth (FWHM) and generally lower CRLB for GABA, Gln, and GSH, along with the previous findings reported in 9.4T studies [45, 56] support the reliability of theses low-concentration metabolite maps obtained here at 7T.

Water images acquired with both fully sampled and accelerated ECCENTRIC exhibit very high correlation coefficients (Suppl. Figs.S2 and S1). While correlation coefficients between ground truth and accelerated ECCENTRIC acquisitions are generally lower for metabolic images (Figs.3–4) than for water images, visual assessments show that the quality of metabolite mapping of healthy brain and tumors is preserved across all accelerations. To explain the discrepancy between results on metabolites and water images, we note that contrary to the water images where correlation coefficients are determined straight from the reconstructed images, the correlation coefficients of metabolic images are influenced also by other pre- and post-reconstruction steps in the processing pipeline such as water removal, fat removal and LCModel fitting. These pre- and post-reconstruction steps introduce additional variability and lower correlation coefficients. In particular, metabolites that are specific for tumor metabolism such as Gly [57] and 2HG [58] have high signal in the tumors but low background outside the tumors. The low background levels of 2HG and Gly outside of tumors have high variability due to noise-like structure, which leads to decreased correlation coefficients for these metabolites when calculated over the entire brain image. However, tumors are still mapped well by these metabolites across all acceleration factors.

The 3D-ECCENTRIC ^1^H-FID-MRSI clinical protocol showed robust performance in glioma patients, where 19 patients had good MRSI quality, and only one patient had low MRSI quality which could not be analyzed due to motion artifacts. The high quality of the data was achieved through the use of third-order shimming, which provides more uniform $B_{0}$ field across the brain, as well as the shortened scan time, which minimized the scanner drift and possibility of subject motion. The scanner drift typically ranged from 5–10 Hz over a 10min scan time.

We showed that the oncometabolite 2HG can be imaged by 3D-ECCENTRIC ^1^H-FID-MRSI at the highest resolution shown to date in newly diagnosed treatment-naive mutant IDH1 glioma patients. Compared to previous high resolution 3D FID-MRSI investigations [21, 53] which exhibited less distinct identification of 2HG in patients with mutant IDH1 glioma, our imaging technique benefited from improved data quality facilitated by third-order shimming and the CS-SENSE-LR reconstruction. Higher-order shimming and reconstruction-integrated $B_{0}$ correction helped improve spectral linewidth, while the low-rank regularized model further enhanced SNR [36, 37]. The increased spectral dispersion at 7T, narrow linewidth (< 0.05 ppm) and high SNR, enhances the ability to fit 2HG in ultra-short TE FID spectra and makes 2HG detection less reliant on particular spectral patterns generated by long TE spectral editing spin-echo sequences [59]. Long TE decrease SNR, and spin echo sequences for spectral editing result in particularly high specific absorption rate at ultra-high field that impose a long TR and prolongs the acquisition time. Previously, we showed that narrow linewidth is the primary experimental factor that determines the precision and accuracy of the 2HG fitting at both 3T and 7T irrespective of spectral editing [59, 60]. High resolution 3D-ECCENTRIC ^1^H-FID-MRSI allows spectral linewidth < 0.05 ppm in glioma tumors. In patients that were treated before the MRSI scan the chance of 2HG detection is significantly reduced by treatment regimens combining surgery, radio and chemotherapy, which are very effective in decreasing the levels of 2HG as shown in previous studies [51, 52]. However, the pre-operative high resolution 2HG imaging has high potential and is valuable for image guided therapy of mutant IDH glioma patients.

In addition to 2HG, several important metabolites for glioma metabolism can be imaged at high resolution, including Glu, Gln, Gly and GSH [57, 61]. In particular, Gln metabolism is an alternative energy source in glioma cells and targeted to induce metabolic vulnerability in synthetic lethality treatment approaches [62]. Furthermore, NAAG has important clinical applications in many brain diseases and our data show the highest resolution for NAAG imaging to date. Our 3D-ECCENTRIC data at 2.5 mm isotropic show unprecedented spatial resolution of 3D MRSI metabolic imaging obtained in glioma patients with an acquisition time of 10min:26s that can still be accommodated in most clinical protocols. At slightly lower voxel size of 3.4 mm isotropic the patients can be imaged in 4min:40s which is 3 times faster than similar methods used in previous patient studies [21, 53].

ECCENTRIC encoding is highly versatile with flexible choice of FoV, spatial resolution, spectral bandwidth that can be set to optimize SNR and acquisition time. The advantage and strength of ECCENTRIC is enabled by the possibility to freely choose the radius and position of circle trajectories in covering the k-space: 1) the free choice of circle radius allows freedom in setting FoV, spatial resolution and spectral bandwidth without the need of temporal interleaving, 2) the free choice of circle position allows freedom for random undersampling the k-space to accelerate acquisition by CS. This flexibility is particularly important for ^1^H-MRSI at 7T and beyond, due to the increased spectral bandwidth required which limits the duration of k-space trajectories. In addition, free choice of circle position should enable FoV with different extent along the axial dimensions for additional time saving, which cannot be achieved by concentric, rosette and spiral trajectories.

The flexibility in setting FoV, resolution and spectral bandwidth by varying the circle radius and CS undersampling to optimize SNR and acquisition time is shown in Supplementary Fig.S4–S6. These results indicate that, when using the same image resolution and acquisition time, ECCENTRIC provides a higher SNR for protocols that use smaller circle radii and higher acceleration compared to protocols using larger circle radii and lower acceleration.

There are some limitations in the current implementation. In particular, reconstruction time of 3D-ECCENTRIC data requires several hours. For example, using a 64 × 64 × 31 matrix size for 3.4 mm isotropic, the water removal step took 1 hour, the CS-SENSE-LR reconstruction took 3 hours on a GPU (or 12 hours on a CPU), and the parallel LCModel fitting took 1 hour on a high-performance server such as the Dell PowerEdge R7525 (with 64 cores of 2.9GHz and 128M cache, 512 GB RAM, and 3 NVIDIA Ampere A40 GPUs). This computation time may be considered relatively long for routine clinical applications. Also, at the moment patient motion or scanner drift is not corrected during ECCENTRIC acquisition, which may increase the variability of metabolite quantification. The metabolite concentrations were not provided in absolute units such as milimolar but expressed in IU relative to the water reference data that still provides comparable values across subjects and scanners. We note that for absolute quantification of ^1^H-FID-MRSI data only the T1 relaxation correction might be needed. Future improvements of our method will seek to shorten reconstruction and provide results faster for clinical use. Also absolute concentrations will be estimated, and the robustness of data acquisition with regard to motion and hardware instability will be increased to reduce variability of metabolite quantification.

In conclusion, we developed ECCENTRIC - an advanced MRSI method at ultra-high field that pushes the spatial and temporal limits of *in vivo* metabolic imaging. ECCENTRIC demonstrated high quality performance in healthy volunteers and patients. We expect that ECCENTRIC will open exciting new avenues in neuroscience by mapping in great detail the brain neurochemistry in healthy and disease conditions to answer important questions and enable discoveries in clinical and research studies.

## Supplementary Material

Supplement 1

## Figures and Tables

**TABLE I:** The demographics of the brain tumor patients along with their histological and molecular diagnoses according to the World Health Organization guidelines (mIDHI = IDH1(R132H) mutation, wtIDH = IDH1/2 wild type). The quality of the MRSI data was rated based on the quality parameters (CRLB< 20%, FWHM< 0.1 ppm, SNR> 5) from spectral fitting by LCModel. Treatment prior to MRSI scan included: PBT = proton beam therapy, IMRT = intensity modulated radiotherapy, VMAT = volumetric modulated arc therapy, SRS = stereotactic radiosurgery, TMZ = temozolomide, HU = hydroxyurea, Bev = bevacizumab. ND = Not Diagnosed by pathology, presumptive diagnosis of glioma is based on imaging features.

Patient #	Age/Gender	Histological Diagnosis		Molecular Diagnosis			Treatment	MRSI quality
		Grade	Type	IDH1 status	1p/19q codel	Other		
01	31/M	2	Astrocytoma	mIDHI	not-deleted	ATRX, TP53	No treatment	Good
02	34/F	3	Astrocytoma	mIDHI	not-deleted		No treatment	Good
03	42/F	2	Astrocytoma	mIDHI	not-deleted		PBT 60Gy	Good
04	42/M	2	Astrocytoma	mIDHI	not-deleted	ATRX	IMRT 60 Gy + C6-TMZ	Good
05	51/M	3	Anaplastic Astrocytoma	mIDHI	not-deleted	ATRX, TP53	Surgery, PBT 60Gy, C6-TMZ	Good
06	66/F	3	Oligodendroglioma	mIDHI	deleted	TP53	No treatment	Good
07	33/F	2	Oligodendroglioma	mIDHI	not-deleted		No treatment	*Bad*
08	28/F	2	Oligodendroglioma	mIDHI	not-deleted		Surgery	Good
09	50/M	2	Oligodendroglioma	mIDHI	not-deleted		Surgery, XRT 60 Gy	Good
10	57/M	2	Oligodendroglioma	mIDHI	deleted		Surgery, C6-TMZ	Good
11	52/F	2	Anaplastic Oligodendroglioma	mIDHI	not-deleted		Surgery, IMRT 60Gy, C11-TMZ	Good
12	62/F	2	Anaplastic Oligodendroglioma	mIDHI	deleted		Surgery, VMAT 60Gy	Good
13	28/M	4	Astrocytoma	mIDHI	not-deleted	MGMT	Surgery, XRT 60 Gy, C6-TMZ	Good
14	37/M	4	Astrocytoma	mIDHI	not-deleted		Surgery, IMRT 60 Gy, C4-TMZ, C5-(TMZ+HU), C9-TMZ	Good
15	57/F	2	Astrocytoma	wtIDH	not-deleted		XRT 60Gy + C6-TMZ	Good
16	35/M	3	Anaplastic Astrocytoma	wtIDH	not-deleted		Surgery, IMRT 60Gy, C18-TMZ, SRS, C4-Bev	Good
17	35/M	4	Glioblastoma	wtIDH	not-deleted	C7/C10, CDK4, EGFR	VMAT 60Gy, C6-TMZ, C2-BEV	Good
18	33/F	ND	Not diagnosed	Not diagnosed	Not diagnosed	Not diagnosed	No treatment	Good
19	32/F	ND	Not diagnosed	Not diagnosed	Not diagnosed	Not diagnosed	No treatment	Good
20	58/M	ND	Not diagnosed	Not diagnosed	Not diagnosed	Not diagnosed	No treatment	Good

**TABLE II:** Metabolite concentrations in institutional units (I.U.) relative to the reference water signal and quantification error estimates by LCModel (Cramér-Rao lower bound, %) in each brain lobe and tissue type. The values are calculated as the average (standard deviation) across the healthy volunteers who were imaged by 3D ECCENTRCIC at 3.4 mm isotropic with AF=2 (9min:20s acquisition time). The two bottom rows in the table present the SNR and FWHM mean (standard deviation) values. The last column shows the percentage of voxels inside the brain and FoV that meet the criteria of acceptable quality: CRLB < 20%, FWHM < 0.07*ppm*, SNR > 5.

Mean across volunteers (Standard deviation)	Frontal		Limbic		Parietal		Occipital		Temporal		usable voxels mean % (std)
	WM	GM	WM	GM	WM	GM	WM	GM	WM	GM	

tCre [I.U.]	3.34 ( 0.34 )	3.84 ( 0.04 )	3.22 ( 0.35 )	4.46 ( 0.28 )	3.30 ( 0.28 )	3.95 ( 0.28 )	3.31 ( 0.30 )	3.39 ( 0.19 )	3.09 ( 0.25 )	3.81 ( 0.19 )	
tCre CRLB [%]	3.25 ( 0.24 )	3.54 ( 0.41 )	3.38 ( 0.31 )	3.18 ( 0.30 )	3.21 ( 0.33 )	3.26 ( 0.26 )	3.45 ( 0.62 )	3.64 ( 0.62 )	3.42 ( 0.37 )	3.33 ( 0.26 )	73.3 (3.3)

NAA [I.U.]	4.16 ( 0.98 )	4.51 ( 0.66 )	4.07 ( 1.01 )	5.38 ( 0.90 )	4.31 ( 0.96 )	4.84 ( 0.88 )	4.33 ( 0.98 )	4.18 ( 0.85 )	3.91 ( 0.84 )	4.35 ( 0.73 )	
NAA CRLB [%]	3.13 ( 0.34 )	3.55 ( 0.64 )	3.26 ( 0.33 )	3.07 ( 0.36 )	2.86 ( 0.26 )	3.05 ( 0.37 )	3.30 ( 0.54 )	3.63 ( 0.64 )	3.32 ( 0.39 )	3.32 ( 0.34 )	73.0 (3.5)

Ins [I.U.]	3.49 ( 0.52 )	3.51 ( 0.35 )	3.65 ( 0.60 )	4.28 ( 0.48 )	3.73 ( 0.66 )	3.58 ( 0.29 )	3.74 ( 0.65 )	3.27 ( 0.57 )	3.56 ( 0.65 )	3.39 ( 0.51 )	
Ins CRLB [%]	5.33 ( 0.64 )	6.04 ( 0.94 )	5.29 ( 0.52 )	5.36 ( 0.42 )	5.16 ( 0.42 )	5.63 ( 0.52 )	5.38 ( 0.48 )	5.84 ( 0.50 )	5.34 ( 0.33 )	5.68 ( 0.40 )	73.0 (3.5)

GPC+PCh [I.U.]	1.03 ( 0.10 )	0.93 ( 0.05 )	1.10 ( 0.10 )	1.24 ( 0.08 )	1.06 ( 0.11 )	0.95 ( 0.09 )	0.94 ( 0.12 )	0.79 ( 0.06 )	0.99 ( 0.07 )	0.98 ( 0.12 )	
GPC+PCh CRLB [%]	3.63 ( 0.34 )	4.24 ( 0.59 )	3.46 ( 0.31 )	3.56 ( 0.27 )	3.56 ( 0.29 )	3.97 ( 0.33 )	4.13 ( 0.57 )	4.59 ( 0.64 )	3.68 ( 0.29 )	3.94 ( 0.25 )	73.5 (3.3)

Glu [I.U.]	3.86 ( 0.53 )	4.50 ( 0.29 )	3.72 ( 0.49 )	5.24 ( 0.46 )	3.86 ( 0.39 )	4.61 ( 0.39 )	3.80 ( 0.36 )	3.82 ( 0.38 )	3.50 ( 0.35 )	4.29 ( 0.44 )	
Glu CRLB [%]	4.51 ( 0.51 )	5.01 ( 0.85 )	4.72 ( 0.59 )	4.37 ( 0.47 )	4.31 ( 0.53 )	4.48 ( 0.55 )	4.82 ( 1.14 )	5.22 ( 1.32 )	4.80 ( 0.74 )	4.71 ( 0.64 )	72.8 (3.8)

Gln [I.U.]	0.89 ( 0.06 )	1.24 ( 0.25 )	0.83 ( 0.06 )	1.33 ( 0.16 )	0.80 ( 0.16 )	1.08 ( 0.26 )	0.87 ( 0.22 )	0.91 ( 0.21 )	0.80 ( 0.09 )	1.07 ( 0.22 )	
Gln CRLB [%]	15.93 ( 2.45 )	14.81 ( 2.22 )	17.72 ( 2.68 )	14.38 ( 2.48 )	17.92 4.78 )	16.22 ( 3.24 )	18.99 5.14 )	18.77 ( 4.11 )	18.30 ( 2.95 )	16.45 ( 2.60 )	56.3 (4.6)

GABA [I.U.]	1.34 ( 0.09 )	1.48 ( 0.29 )	1.34 ( 0.10 )	1.79 ( 0.37 )	1.37 ( 0.14 )	1.58 ( 0.30 )	1.33 ( 0.18 )	1.37 ( 0.18 )	1.21 ( 0.16 )	1.39 ( 0.32 )	
GABA CRLB [%]	8.68 ( 1.10 )	9.61 ( 1.51 )	8.86 ( 1.50 )	8.52 ( 1.74 )	8.54 ( 2.08 )	8.78 ( 1.76 )	9.48 ( 2.34 )	9.90 ( 2.22 )	9.60 ( 2.16 )	9.52 ( 2.09 )	68.3 (1.5)

GSH [I.U.]	1.07 ( 0.24 )	1.22 ( 0.05 )	1.11 ( 0.26 )	1.45 ( 0.20 )	1.10 ( 0.20 )	1.17 ( 0.07 )	1.05 ( 0.18 )	1.00 ( 0.09 )	1.02 ( 0.17 )	1.14 ( 0.12 )	
GSH CRLB [%]	8.20 ( 0.88 )	8.77 ( 1.15 )	8.29 ( 0.93 )	7.96 ( 0.79 )	8.04 ( 1.00 )	8.43 ( 0.87 )	9.12 ( 2.01 )	9.80 ( 1.90 )	8.65 ( 1.23 )	8.81 ( 1.17 )	71.5 (3.1)

NAAG [I.U.]	0.82 ( 0.14 )	0.39 ( 0.12 )	0.90 ( 0.13 )	0.49 ( 0.17 )	0.96 ( 0.17 )	0.34 ( 0.13 )	0.78 ( 0.16 )	0.41 ( 0.12 )	0.76 ( 0.11 )	0.35 ( 0.10 )	
NAAG CRLB [%]	14.11 ( 4.10 )	19.02 ( 5.07 )	12.43 ( 3.87 )	17.35 ( 6.07 )	12.01 ( 3.14 )	18.03 ( 5.15 )	15.51 ( 5.08 )	19.40 ( 6.62 )	15.36 ( 5.01 )	20.13 ( 6.40 )	45.3 (11.6)

SNR	24.64 ( 4.41 )	23.67 ( 3.84 )	22.92 ( 4.50 )	24.53 ( 4.02 )	25.64 ( 4.82 )	24.73 ( 4.17 )	22.48 ( 6.13 )	21.32 ( 5.56 )	22.07 ( 4.23 )	22.40 ( 3.37 )	∅

FWHM [Hz]	11.43 ( 0.91 )	12.09 ( 0.77 )	11.28 ( 1.15 )	11.13 ( 1.13 )	10.21 0.79 )	11.02 ( 0.84 )	11.64 ( 0.91 )	12.33 ( 1.04 )	12.26 ( 1.23 )	12.38 ( 1.22 )	∅
