# Supplementary material for: ECCENTRIC: a fast and unrestrained approach for high-resolution in vivo metabolic imaging at ultra-high field MR
Source: ArXiv. 2023 Dec 21:arXiv:2305.13822v2. Originally published 2023 May 23. Preprint. [Version 2] (PMC10246065)
Supplement: 1 [file NIHPP2305.13822V2-supplement-1.pdf]

VI. SUPPLEMENTARY MATERIAL : ADDITIONAL DATA

VII. ADDITIONAL DATA

A. ECCENTRIC water imaging in healthy volunteers

To investigate the ability of ECCENTRIC to image brain structure, we performed water imaging in several healthy volunteers using 3D FID-MRSI ECCENTRIC. The water suppression was turned off, and we used the 3.4 mm protocol with larger FA = 40° and shorter TR = 100 ms to produce T_1 weighed images. ECCENTRIC k-space data were acquired fully sampled (AF=1) and the acceleration was obtained by retrospective CS undersampling in post-processing. Results in Fig. S1 show that fully sampled ECCENTRIC images reveal similar brain structure as T_1-weighted GRE images acquired with matched spatial resolution and tissue contrast. Considering the fully sampled (AF=1) ECCENTRIC as ground truth, the SSIM $\geq$ 0.99 and correlation factor $\geq$ 0.92 for images obtained with AF = 1-12. Visually, almost no difference can be observed between images obtained with AF = 1-4. For AF $\geq$ 8 the noise level increases, which interferes with fine structural details.

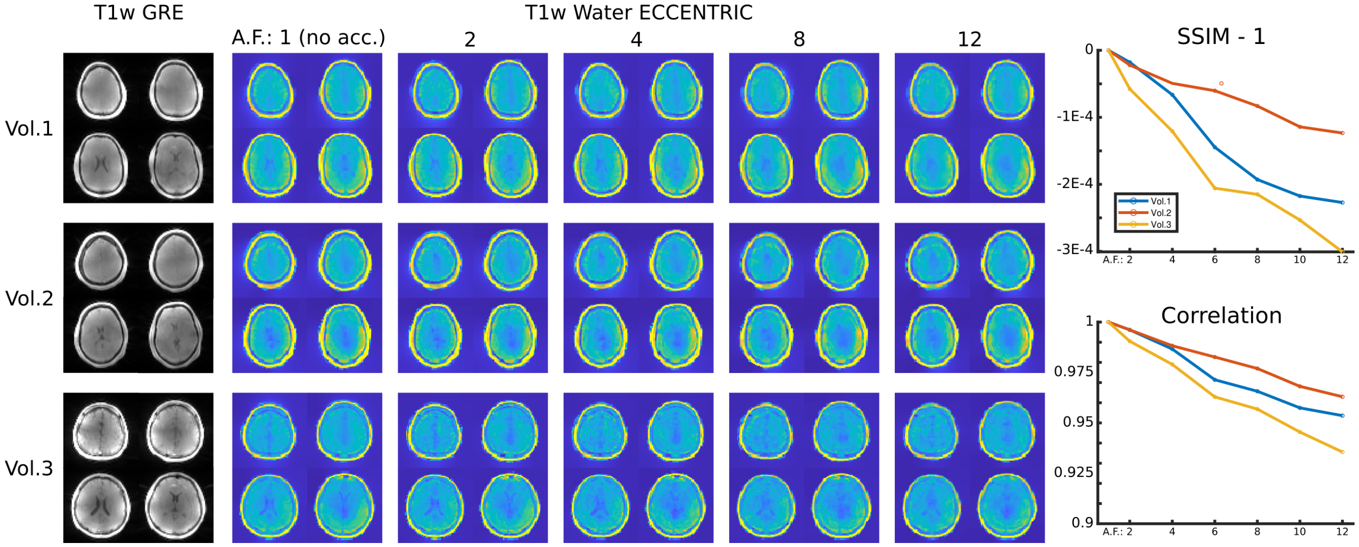

FIG. S1: Water imaging of human brain in healthy volunteers using 3D-ECCENTRIC with 3.4mm isotropic voxel size. Left, images obtained by T_1-weighted 3D GRE acquired with matched FA, TR, TE and spatial resolution. Middle, water images obtained with 3D-ECCENTRIC for acceleration factors 1-12. Right, SSIM and correlation factors for accelerated ECCENTRIC water images are calculated considering the fully sampled image (AF=1) as ground truth. Four slices are shown for each volunteer.

VIII. OPTIMIZING ECCENTRIC FOR HIGH SNR AND ACCELERATED HIGH-RESOLUTION MRSI

By design, the k-space acquisition by ECCENTRIC is characterized by: the circle radius (CR), compress sensing acceleration factor (AF), image matrix size (MS) and field-of-view (FoV). In addition, acquisition of the time dimension

(FID) for spectroscopy is characterized by the spectral bandwidth (SW), the dwell-time, and the number of time points. In particular, for spectral-spatial encoding (SSE) there is a dependency between the spectral bandwidth, field-of-view and image resolution. Compared to other SSE schemes, ECCENTRIC allows very high flexibility in the choice of SW, FoV and MS, which is particularly needed at ultra-high field (7T and beyond) and to operate within the technical limits of the gradient system minimizing electrical, mechanical and thermal stress.

Importantly, the ECCENTRIC flexibility can be used to optimize the SNR and acquisition time while pushing the image resolution. ECCENTRIC parameters have different impact on the measured signal-to-noise ratio (SNR) and acquisition time (TA) as highlighted in the following table:

Change \ Effect	SNR	TA
Circle Radius $\nearrow$	$\searrow$	$\searrow$
CS Acceleration $\nearrow$	$\rightarrow$ (image smoothness $\nearrow$)	$\searrow$
Matrix Size $\nearrow$	$\searrow$	$\nearrow$

TABLE S1

3D ECCENTRIC MRSI can be optimized by reducing CR and thus increasing the sampling density in k-space, which can be designed to sample more the center of k-space to increase SNR. In addition, the CR reduction allows a large range of spectral windows by controlling the gradient slew rate as needed based on the image resolution. However, the reduction in CR requires a higher CS acceleration for an equivalent acquisition time. As we showed, CS acceleration up to 4 provides high quality metabolite images, and this can be traded to optimize SNR with smaller CR.

To explore the flexibility of ECCENTRIC parameters for SNR optimization, three acquisitions with the same isotropic resolution (5mm) and acquisition time (14min) were acquired with different ECCENTRIC circle radii (CR) and CS acceleration factors: 1) CR = kmax/4 and AF = 1; 2) CR = kmax/8 and AF = 2; 3) CR = kmax/16 and AF = 4. Sampling patterns of the k-space are shown in Fig. S2.

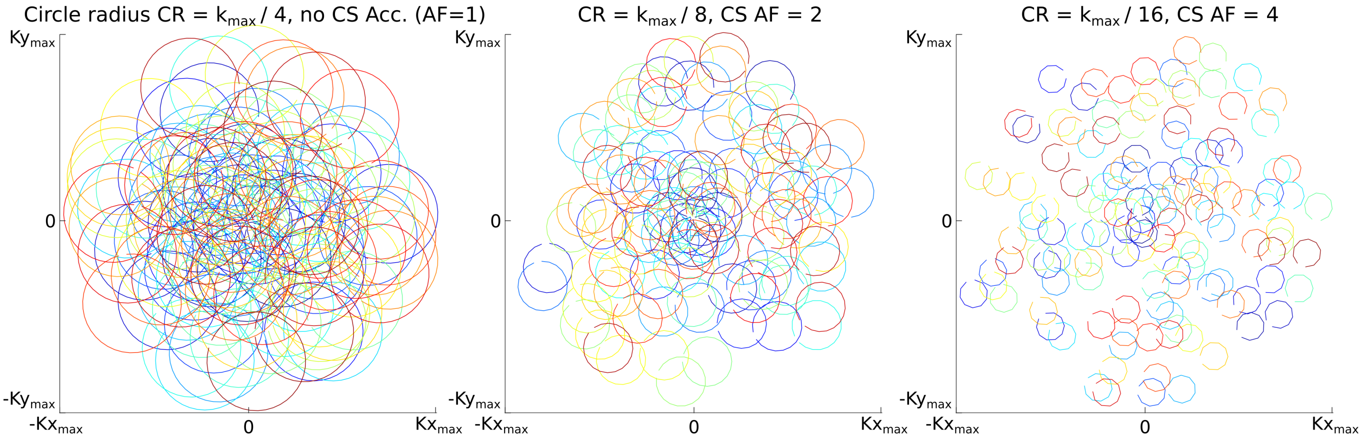

FIG. S2: ECCENTRIC k-space sampling for various circle radii (CR) and compress sensing acceleration (AF).

Metabolite maps obtained in a healthy volunteer are presented in Fig. S3. Higher SNR can be noticed for the six metabolites as the circle radii is decreased, while only minor blurring is apparent at the highest acceleration.

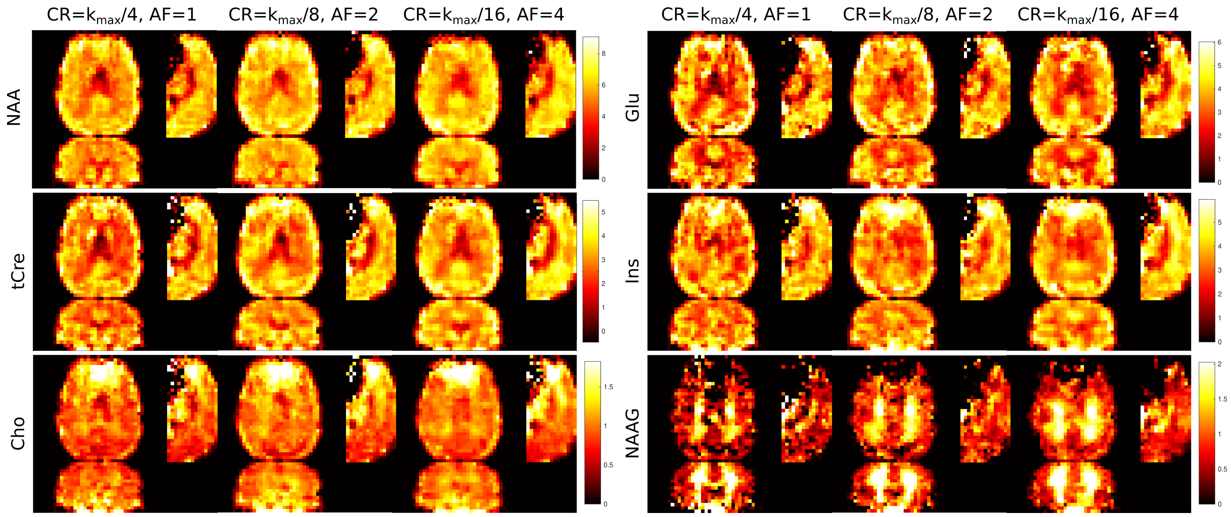

FIG. S3: Metabolite maps of NAA, total Creatine, total Choline, Glu, Ins and NAAG produced from 3D ^{1}H-FID-MRSI ECCENTRIC acquisitions at 5 mm isotropic image resolution in 14 min with various circle radii CR and compress sensing AF.

Quantitative analysis in Fig. S4 shows that decreasing CR results in a notable gain in metabolite SNR of + 30% for kmax/8 and + 40% for kmax/16 relative to kmax/4, respectively. In the same time, the linewidth is stable across the different protocols. The increase in SNR enables more precise metabolite quantification resulting in lower CRLB for spectral fitting, especially for the low signal metabolites. Among the 3 protocols, the protocol with CR = kmax/8 and AF = 2 showed the best performance with a marked increase in SNR and little visible blurring on metabolite maps. These results demonstrate that CR and AF allow SNR optimization of 3D ECCENTRIC MRSI for a desired image resolution and acquisition time.

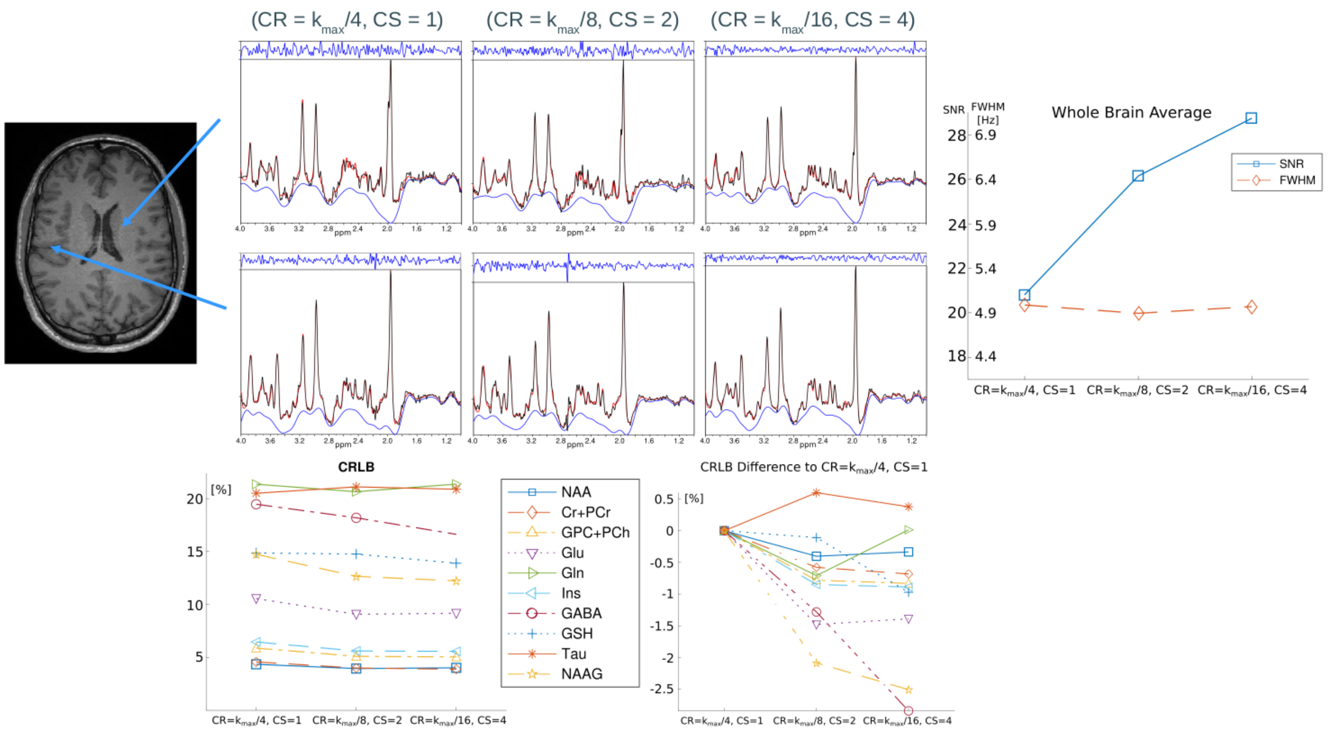

FIG. S4: Quantitative analysis of 3D ^{1}H-FID-MRSI ECCENTRIC acquisitions with various circle radius CR and compress sensing AF.
