# Supplementary material for: ECCENTRIC: a fast and unrestrained approach for high-resolution in vivo metabolic imaging at ultra-high field MR
Source: ArXiv. 2023 May 23:arXiv:2305.13822v1. Preprint. [Version 1] (PMC10246065)
Supplement: Supplementary file 1 [file NIHPP2305.13822v1-supplement-1.pdf]

VII. SUPPLEMENTARY MATERIAL TO *ECCENTRIC: A FAST AND UNRESTRAINED APPROACH FOR HIGH-RESOLUTION IN VIVO METABOLIC IMAGING AT ULTRA-HIGH FIELD MR*

A. Additional data

1. ECCENTRIC imaging on high-resolution phantom

To evaluate the performance of ECCENTRIC sampling, we initially tested it on a high-resolution structural-metabolic phantom using a 2D FID-MRSI sequence. We used a custom made phantom with geometry similar to Derenzo molecular imaging phantom [63] containing 5 sets of tubes with diameters of 2, 4, 6, 8 and 10 mm as shown in Fig.S1). Each set contained 6 tubes of identical diameter separated by a distance equal to twice the inner diameter positioned in a triangular configuration. In every set, the six tubes were filled with metabolite solutions containing 10 mM of creatine. Magnevist (Gd-DTPA) was added (1 mL/L) in each tube to shorten T_1 and create T_1-weighted contrast for structural MRI. The whole tube structure was inserted in a large cylindrical container (13.33 cm inner diameter) which was filled with 10 mM NaCl solution. Further details of phantom manufacturing and chemical composition are mentioned in [37].

Because the phantom has geometric structure only in the axial section we acquired 2D-ECCENTRIC over a single slice of 10 mm thickness. The 2D-ECCENTRIC sampling was the same like the central k-space partition from 3D-ECCENTRIC. The 2D-ECCENTRIC was acquired using the same RF-pulse and FA, TE of 1.15 ms and TR of 450 ms to account for the longer T_1 relaxation times in the phantom. The FID was measured with a bandwidth of 2000 Hz over 350 ms, and successive acquisitions were performed with increased in-plane resolution. The circle radius (R) was set to 1/8, 1/8, 1/9, and $1/10\frac{M}{FoV}$ for 4.6, 3.4, 2.8, and 2.0 mm in-plane resolutions, respectively, to avoid temporal interleaving for any spatial resolution. In addition to metabolite imaging, to better assess the performance of ECCENTRIC for spatial encoding we also performed water imaging of the structural phantom at the same spatial resolutions as metabolites. To achieve this, the 2D ECCENTRIC sequence without water suppression was utilized with a short TR of 100 ms and an FA of 40 degrees to maximize T_1-weighted (T_1w) contrast of the tubes inside the cylindrical phantom. Following this, the first point of the acquired timeseries was reconstructed to generate the T_1-weighted water image. Both metabolite and water data were acquired with fully sampled ECCENTRIC. To investigate accelerations, the fully sampled ECCENTRIC data were retrospectively undersampled for acceleration factors (AF) between $2 - 12$.

The effect of the acceleration on water and metabolite imaging was evaluated by analyzing the structural similarity index (SSIM) and correlation coefficient for all voxels inside the phantom with respect to the fully sampled data.

Water imaging of a single slice showed that 2D ECCENTRIC can resolve the structural details of the phantom up to the resolution targeted by the imaging protocol (Fig. S1). Compared to the fully sampled acquisition (AF=1), no visible difference in image quality can be seen for retrospective accelerations factors between 1-4, minor changes can be detected for AF between 4-8, and moderate loss of details for AF 8-12. Considering AF=1 as ground truth, quantitative analysis reveals that SSIM $\geq$ 0.99 across all resolutions for accelerations up to AF = 4, and SSIM decreases to 0.97 for the highest acceleration and resolution tested. Similarly, correlation factors larger than 0.99 are observed up AF = 4, which decrease to 0.95 for the lowest resolution and largest acceleration factor. Comparing the different spatial resolutions, CS acceleration shows better performance for smaller voxel size.

For metabolite imaging, the tCr metabolite was imaged with 2D ECCENTRIC for the same resolutions and acceleration factors as in the water imaging (Fig. S1). Similar results are observed: 1) for acceleration factors between 1-4 metabolite maps show similar quality, while 2) for larger acceleration factors there is a moderate loss of image details and and increase in the noise level. Considering AF=1 as ground truth, across the entire series of measurements SSIM range between 0.75-0.89, and correlation factors between 0.79-0.96. In contrast to the water imaging, the highest SSIM and correlation factors are not exhibited at the smallest voxel size for accelerated metabolic imaging due to lower SNR of metabolites compared to water.

In particular, we note that for isotropic voxel size of 3.4 mm and for acceleration factors up to 4 we obtained the highest SSIM and correlation factors for metabolic imaging. Hence, we concluded that ECCENTRIC at 3.4 mm with AF 1-4 represents a good starting protocol to further investigate fast high resolution metabolic imaging of the human brain.

2. ECCENTRIC water imaging in healthy volunteers

To assess the validity of ECCENTRIC acquisition with CS-SENSE-LR reconstruction, three healthy volunteers were scanned with a T_1w 3D water acquisition. The same 3D-ECCENTRIC ^{1}H-FID-MRSI sequence described earlier (3.4 mm isotropic) was used but with a short TR of 100 ms, a higher FA of 40 degrees, and without water suppression.

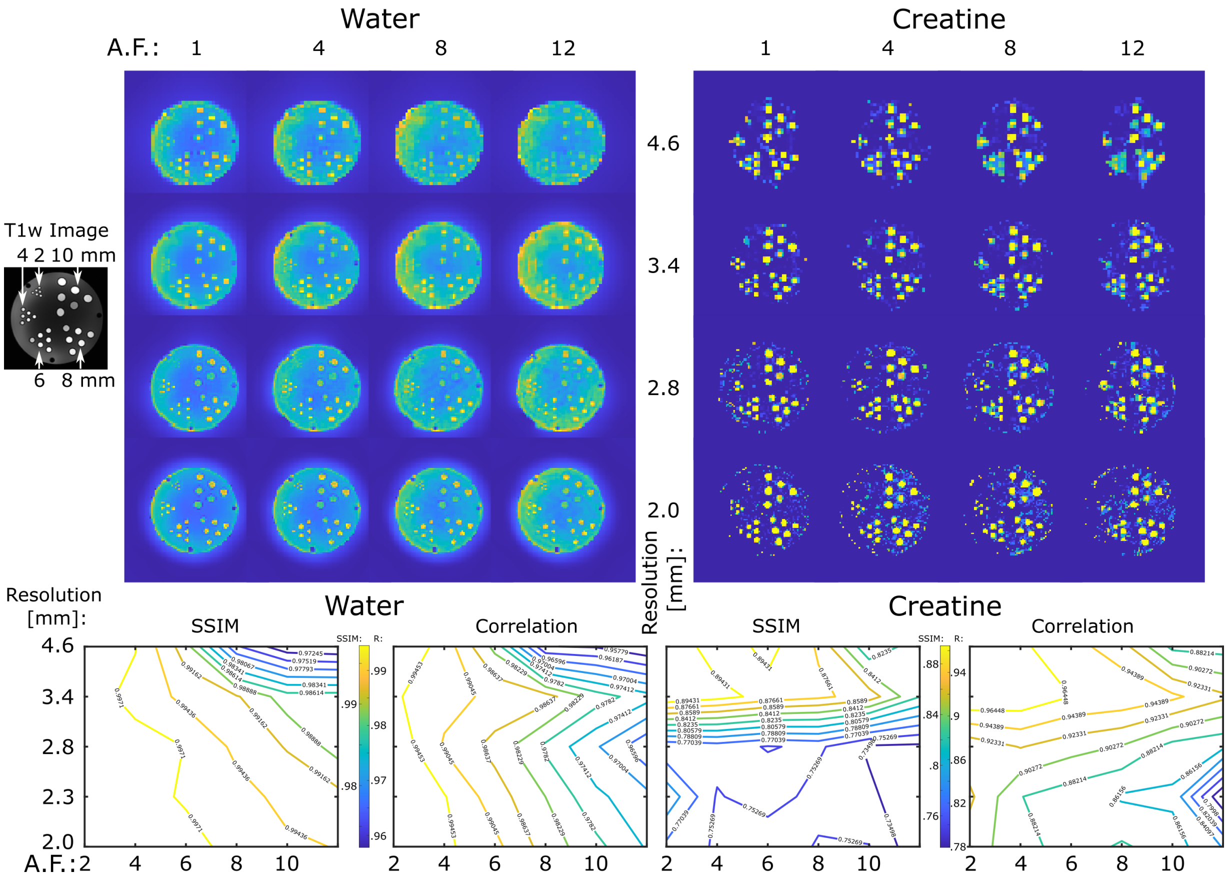

FIG. S1: ECCENTRIC imaging of water and metabolites in the high-resolution structural-metabolic phantom. ECCENTRIC performance was tested for spatial resolutions of 4.6, 3.4, 2.8, 2.0 mm and acceleration factors between 1-12. Top, examples of water and creatine images are shown for all 4 resolutions and 4 AF: 1,4,8,12. Bottom, SSIM and correlation factors for each resolution and acceleration are calculated considering as ground truth the fully sampled image (AF=1).

Reconstructed images were obtained using the model of Eq. 2, and only the first point of the FID was used. To test the acceleration performance, we retrospectively undersampled the T_1w 3D-ECCENTRIC data by AF 2, 4, 6, 8, 10, and 12. For qualitative comparison, a T_1w Cartesian gradient-echo (GRE) imaging sequence was also scanned on the volunteers with matched spatial resolution, TR and TE. The performance of the accelerated ECCENTRIC acquisition and CS-SENSE-LR reconstruction method was assessed with the structural similarity index (SSIM) and the correlation coefficient of all voxels inside the brain relative to the non-accelerated ECCENTRIC (Fig. S2). To investigate the ability of ECCENTRIC to image brain structure, we performed water imaging in several healthy volunteers using 3D FID-MRSI ECCENTRIC. The water suppression was turned off, and we used the 3.4 mm protocol with larger FA = 40° and shorter TR = 100 ms to produce T_1 weighed images. ECCENTRIC k-space data were acquired fully sampled (AF=1) and the acceleration was obtained by retrospective CS undersampling in post-processing. Results in Fig. S2 show that fully sampled ECCENTRIC images reveal similar brain structure as T_1-weighted GRE images acquired with the matched spatial resolution and tissue contrast. Considering the fully sampled (AF=1) ECCENTRIC as ground truth, the SSIM $\geq$ 0.99 and correlation factor $\geq$ 0.92 for images obtained with AF = 1-12. Visually, almost no difference can be observed between images obtained with AF = 1-4. For AF $\geq$ 8 the noise level increases, which interferes with fine structural details.

3. Stability and reproducibility of ECCENTRIC metabolic imaging

Stability and reproducibility of ECCENTRIC was investigated by test-retest of repeated imaging in four healthy volunteers. 3D ECCENTRIC ^{1}H-FID-MRSI was acquired at 3.4 mm isotropic resolution in four consecutive scans using AF=1 (18min:40s), AF=2 (9min:20s), AF=3 (6min:16s), and AF=4 (4min:40s).

Results are shown in Fig.S3 for Glu imaging. Due to high concentration of Glu in gray mater, Glu images have high

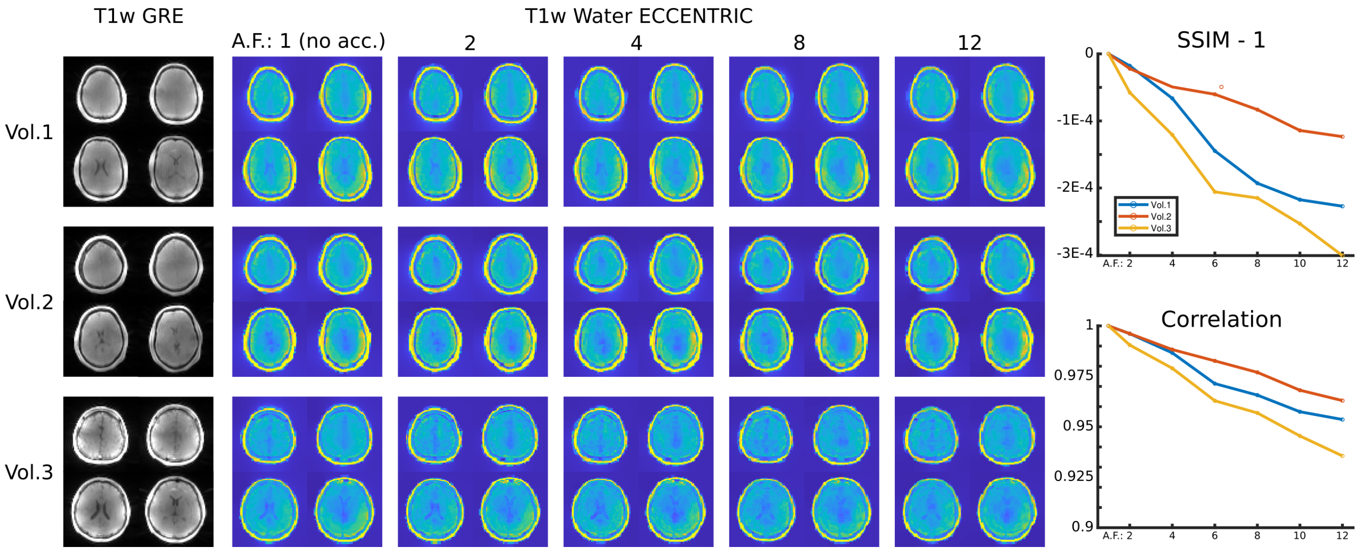

FIG. S2: Water imaging of human brain in healthy volunteers using 3D-ECCENTRIC with 3.4mm isotropic voxel size. Left, images obtained by T_1-weighted 3D GRE acquired with matched FA, TR, TE and spatial resolution. Middle, water images obtained with 3D-ECCENTRIC for acceleration factors 1-12. Right, SSIM and correlation factors for accelerated ECCENTRIC water images are calculated considering the fully sampled image (AF=1) as ground truth.

gray-white matter contrast and show fine structural details of brain that can be used to visually assess the stability and reproducibility of test-retest imaging. It can be seen that across all four scans in all four subjects the metabolite images appear visually similar. We note that with repeated measurements some anatomical differences may also be attributed to slight head motion.

Detailed analysis of the reproducibility of metabolites measurement in healthy volunteers is presented in Tables S1 and S2. The metabolite data from three consecutive measurements with AF=3 performed in the 4 subjects were used to calculate inter-measurement (Table S1) and inter-subject (Table S2) coefficients-of-variation (COV).

	Inter-measurement COV									
	Frontal		Limbic		Parietal		Occipital		Temporal	
	WM	GM	WM	GM	WM	GM	WM	GM	WM	GM
NAA	0.06	0.03	0.06	0.05	0.06	0.05	0.05	0.07	0.06	0.06
tCr	0.05	0.05	0.05	0.03	0.03	0.04	0.04	0.03	0.06	0.05
Ins	0.05	0.04	0.07	0.06	0.05	0.05	0.04	0.06	0.06	0.07
GPC+PCh	0.04	0.03	0.03	0.04	0.04	0.06	0.03	0.04	0.03	0.05
Glu	0.05	0.03	0.05	0.05	0.07	0.07	0.07	0.07	0.06	0.07
Gln	0.14	0.10	0.15	0.11	0.12	0.12	0.12	0.07	0.14	0.08
GABA	0.07	0.11	0.05	0.07	0.09	0.12	0.05	0.11	0.06	0.11
GSH	0.12	0.10	0.14	0.13	0.13	0.13	0.13	0.10	0.14	0.11
NAAG	0.23	0.41	0.28	0.33	0.24	0.55	0.26	0.40	0.28	0.54

TABLE S1: The inter-measurement coefficient of variation (COV) for each metabolite was determined in every lobe and tissue type for the 3D-ECCENTRIC ^{1}H-FID-MRSI acquired at 3.4 mm isotropic resolution in 6min:16sec (AF=3). The inter-measurement COV was calculated as the standard deviation divided by the mean of three consecutive measurements for each volunteer and then averaged across four volunteers. The three AF=3 datasets were obtained from measurements performed with different accelerations (as shown in Fig. S3), but retrospectively undersampled by a factor of 3 for AF=1 and 3/2 for AF=2.

Inter-measurement COV smaller than 7% are observed for five metabolites (NAA, creatine, myo-inositol, choline, glutamate) that are the most abundant in the brain. COV between $8\% - 14\%$ are obtained for glutamine, glutathione and GABA. NAAG has higher COV in brain regions (gray matter) where its concentration is low.

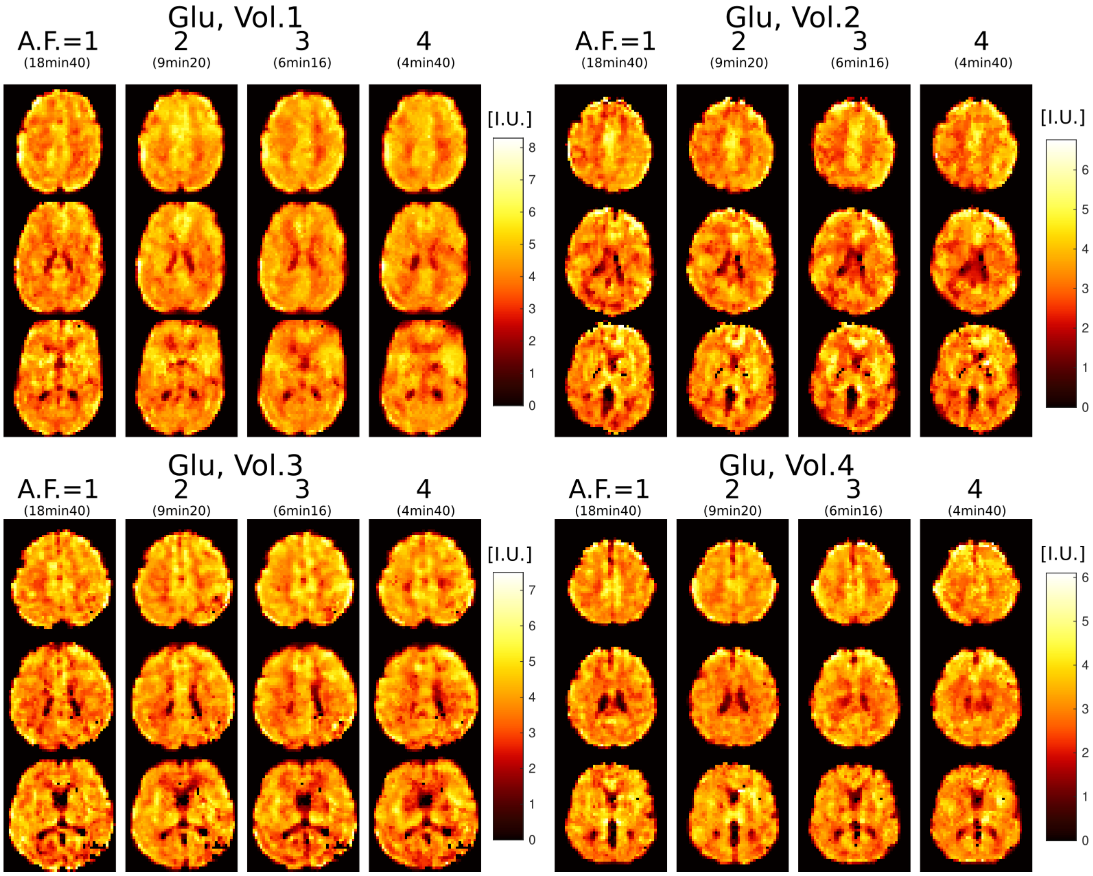

FIG. S3: Glu imaging at 3.4mm isotropic voxel size in four healthy volunteers scanned with 3D-ECCENTRIC ^{1}H-FID-MRSI in four successive acquisitions with increasing accelerations AF=1, 2, 3 and 4. Three slices are shown for each volunteer at each acceleration.

	Inter-subject COV									
	Frontal		Limbic		Parietal		Occipital		Temporal	
	WM	GM	WM	GM	WM	GM	WM	GM	WM	GM
NAA	0.21	0.15	0.23	0.17	0.23	0.17	0.24	0.23	0.23	0.22
Cr+PCr	0.10	0.05	0.12	0.07	0.11	0.05	0.09	0.09	0.11	0.09
Ins	0.18	0.15	0.22	0.17	0.22	0.14	0.21	0.26	,0.24	0.19
GPC+PCh	0.06	0.07	0.07	0.06	0.10	0.10	0.12	0.14	0.11	0.15
Glu	0.19	0.13	0.20	0.16	0.20	0.15	0.18	0.18	0.18	0.20
Gln	0.16	0.25	0.12	0.16	0.18	0.21	0.21	0.23	0.17	0.18
GABA	0.37	0.33	0.39	0.38	0.36	0.36	0.38	0.39	0.37	0.40
GSH	0.13	0.09	0.15	0.11	0.13	0.07	0.11	0.13	0.11	0.12
NAAG	0.32	0.37	0.35	0.29	0.45	0.47	0.50	0.48	0.38	0.29

TABLE S2: The inter-subject coefficient of variation (COV) for each metabolite was determined in every lobe and tissue type for the 3D-ECCENTRIC ^{1}H-FID-MRSI acquired at 3.4 mm isotropic resolution in 6min:16sec (AF=3). The inter-subject COV was calculated as the standard deviation divided by the mean of 3 scanned volunteers and then averaged across the three measurements. The three AF=3 datasets were obtained from measurements performed with different accelerations(AF=1, AF=2 and AF=3), where AF=1 data was retrospectively undersampled by a factor of 3 and AF=2 was retrospectively underssampled by a factor of 3/2.

The inter-subject COV in (Table S2) reflects the biological variability of metabolite concentrations in the gray and white matter across the group of subjects.

3D-ECCENTRIC ^{1}H-FID-MRSI had reproducible performance in three repeat measurements. The method variability (inter-measurement COV) due to the technical performance of 3D-ECCENTRIC ^{1}H-FID-MRSI (Table S1) is much lower (2-4 times smaller) than the biological variability (inter-subject COV) of brain metabolism across individuals (Table S2). The low variability of ECCENTRIC in repeat measurements indicates high precision of metabolite quantification and high potential for longitudinal studies to detect metabolite changes due to disease, treatment and functional tests. The quantification of the five main metabolites that have the highest SNR in brain MRSI (NAA, tCre, Cho, Ins, Glu) shows the lowest variability, with a slight increase in the case of less abundant metabolites (Gln, GABA and GSH). The highest variability is noticed for NAAG outside of the fronto-parietal white matter due to its specific localization in this brain area. We note that in-vivo variability of metabolite quantification in repeat measurements is also influenced by patient motion and scanner stability in addition to 3D-ECCENTRIC MRSI, hence methods that reduce the effects of motion and drift [64, 65] are likely to reduce variability.

B. Optimizing ECCENTRIC for high SNR and accelerated high-resolution MRSI

By design, the k-space acquisition by ECCENTRIC is characterized by: the circle radius (CR), compressed sensing acceleration factor (AF), image matrix size (MS) and field-of-view (FoV). In addition, acquisition of the time dimension (FID) for spectroscopy is characterized by the spectral bandwidth (SW), the dwell-time, and the number of time points. In particular, for spectral-spatial encoding (SSE) there is a dependency between the spectral bandwidth, field-of-view and image resolution. Compared to other SSE schemes, ECCENTRIC allows very high flexibility in the choice of SW, FoV and MS, which is particularly needed at ultra-high field (7T and beyond) and to operate within the technical limits of the gradient system minimizing electrical, mechanical and thermal stress.

Importantly, the ECCENTRIC flexibility can be used to optimize the SNR and acquisition time while pushing the image resolution. ECCENTRIC parameters have different impact on the measured signal-to-noise ratio (SNR) and acquisition time (TA) as highlighted in the following table:

Change \ Effect	SNR	TA
Circle Radius ↗	↘	↘
CS Acceleration ↗	→ (image smoothness ↗)	↘
Matrix Size ↗	↘	↗

TABLE S3:

3D ECCENTRIC MRSI can be optimized by reducing CR and thus increasing the sampling density in k-space, which can be designed to sample more the center of k-space to increase SNR. In addition, the CR reduction allows a large range of spectral windows by controlling the gradient slew rate as needed based on the image resolution. However, the reduction in CR requires a higher CS acceleration for an equivalent acquisition time. As we showed, CS acceleration up to 4 provides high quality metabolite images, and this can be traded to optimize SNR with lower CR.

To explore the flexibility of ECCENTRIC parameters for SNR optimization, three acquisitions with the same isotropic resolution (5mm) and acquisition time (14min) were acquired with different ECCENTRIC circle radii (CR) and CS acceleration factors: 1) CR = kmax/4 and AF = 1; 2) CR = kmax/8 and AF = 2; 3) CR = kmax/16 and AF = 4. Sampling patterns of the k-space are shown in Fig. S4.

Metabolite maps obtained in a healthy volunteer are presented in Fig. S5. Higher SNR can be noticed for the six metabolites as the circle radii is decreased, while only minor blurring is apparent at the highest acceleration.

Quantitative analysis in Fig. S6 shows that decreasing CR results in a notable gain in metabolite SNR of + 30% for kmax/8 and + 40% for kmax/16 relative to kmax/4, respectively. In the same time, the linewidth is stable across the different protocols. The increase in SNR enables more precise metabolite quantification resulting in lower CRLB for spectral fitting, especially for the low signal metabolites. Among the 3 protocols, the protocol with CR = kmax/8 and AF = 2 showed the best performance with a marked increase in SNR and little visible blurring on metabolite maps. These results demonstrate that CR and AF allow SNR optimization of 3D ECCENTRIC MRSI for a desired image resolution and acquisition time.

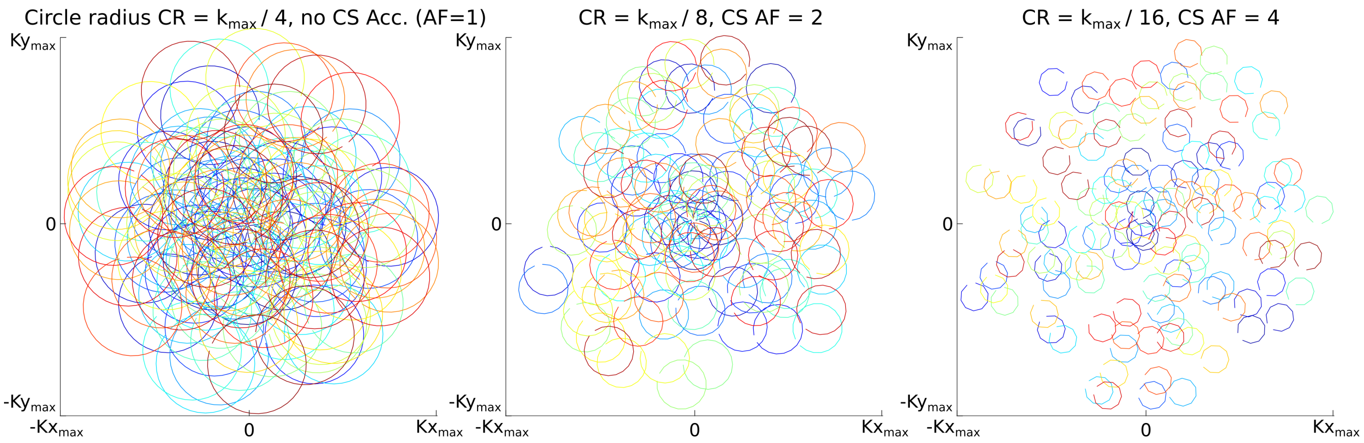

FIG. S4: ECCENTRIC k-space sampling for various circle radii (CR) and compressed sensing acceleration (AF).

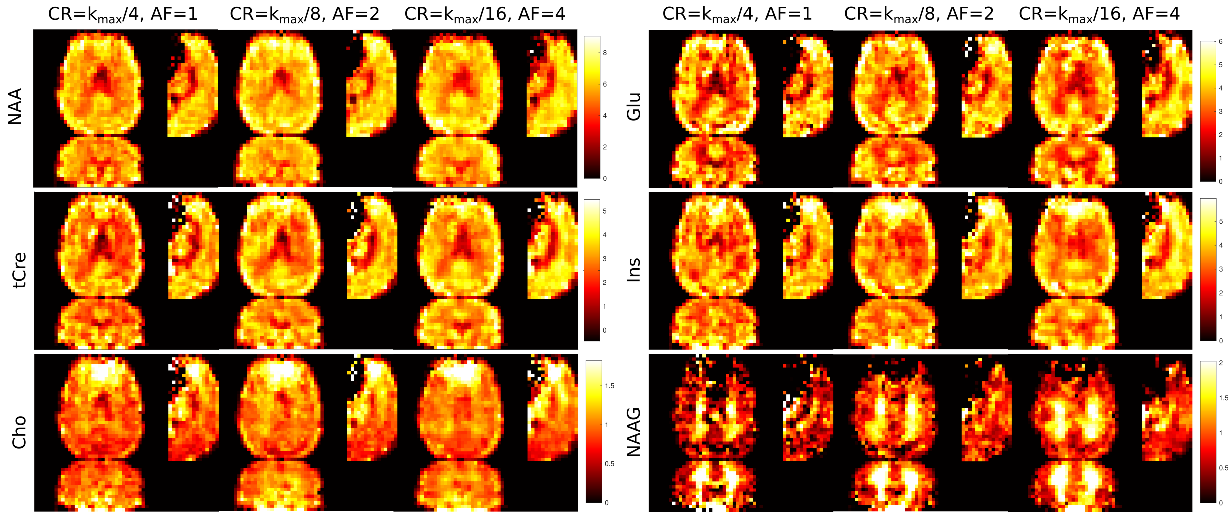

FIG. S5: Metabolite maps of NAA, total Creatine, total Choline, Glu, Ins and NAAG produced from 3D ^{1}H-FID-MRSI ECCENTRIC acquisitions at 5 mm isotropic image resolution in 14 min with various circle radii CR and compressed sensing AF.

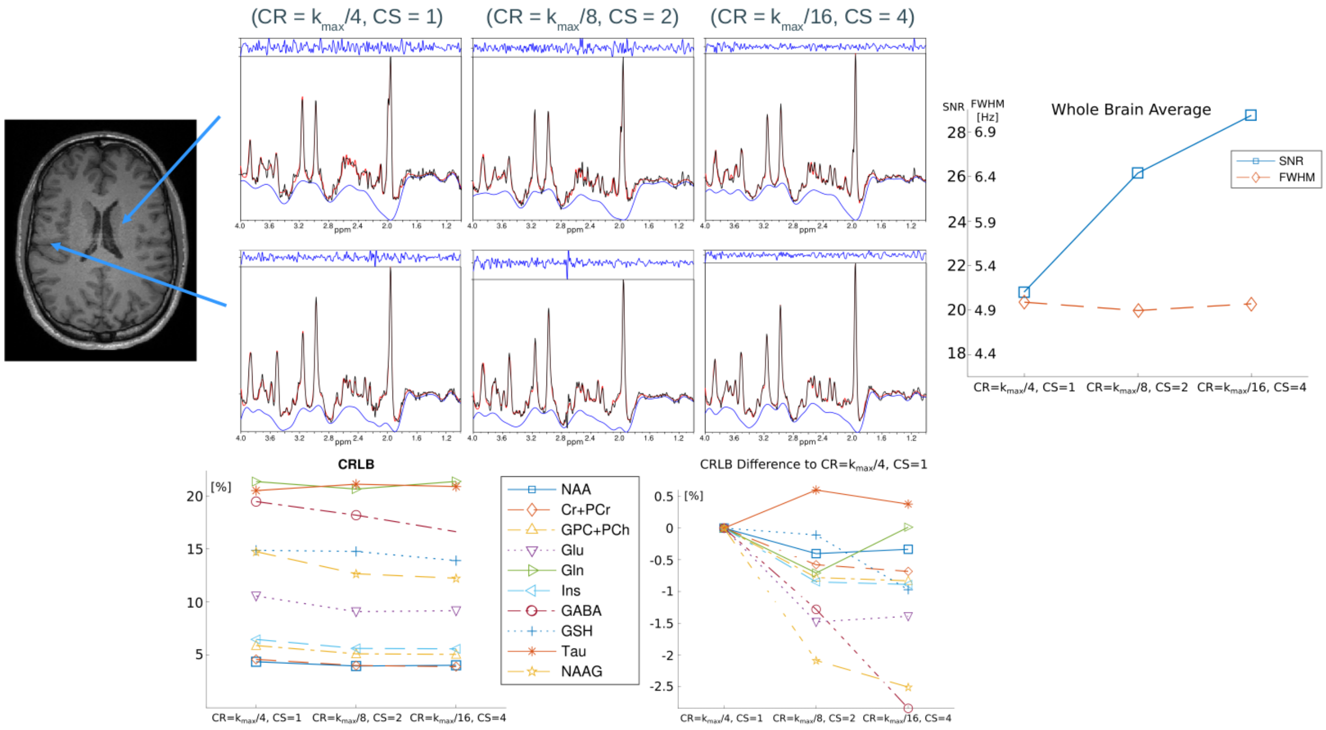

FIG. S6: Quantitative analysis of 3D-ECCENTRIC ^{1}H-FID-MRSI acquisitions with various circle radius CR and compressed sensing AF.
